# Photothermally Driven Ultrafast Polymerase Chain Reaction: Mechanisms, Nanomaterial Architectures, and System Integration

**DOI:** 10.34133/research.0839

**Published:** 2025-08-15

**Authors:** Yile Fang, Lijun Cai, Ning Li, Feika Bian, Dagan Zhang, Nongyue He, Zhiyang Li, Hong Yan, Yuanjin Zhao

**Affiliations:** ^1^ Department of Clinical Laboratory, Institute of Translational Medicine, Nanjing Drum Tower Hospital, Affiliated Hospital of Medical School, Nanjing University, Nanjing 210008, China.; ^2^ State Key Laboratory of Digital Medical Engineering, School of Biological Science and Medical Engineering, Southeast University, Nanjing 210096, China.; ^3^ Laboratory Medicine Center, the Second Affiliated Hospital of Nanjing Medical University, Nanjing 210011, China.

## Abstract

As one of the most important technologies in molecular biology, polymerase chain reaction (PCR) has been widely recognized in many fields such as infectious disease diagnosis due to its high sensitivity, specificity, and accuracy. Attempts in this field are focused on developing efficient heating mechanism to achieve efficient thermal cycles. Recently, with the in-depth research into photothermal effects, photonic PCR technology based on photothermal nanomaterials has gradually demonstrated potential to develop a new generation of ultrafast PCR instrument. Herein, we first categorize the various photothermal nanomaterials and briefly introduce their photothermal conversion mechanisms. Then, we review the photonic PCR technologies based on different nanomaterials and various heating strategies, comparing their advantages and disadvantages. We also discuss the application of photonic PCR in point-of-care testing (POCT) of nucleic acid and summarize the prospects and challenges of photonic PCR technology in clinical diagnostic applications. Finally, we look forward to the promising future research focus of photonic PCR. With this review, researchers can get a comprehensive understanding of photonic PCR from the aspects of technical principles, material selection, equipment development strategies, and so on, paving the way for future research.

## Introduction

Infectious diseases are considered as one of the major threats to human survival. Over the last 20 years, various infectious diseases have emerged, such as severe acute respiratory syndrome (SARS), influenza virus, Middle East respiratory syndrome (MERS), Ebola, and the best known COVID-19, which have caused enormous loss of life and property worldwide [1–4]. The pandemics of infectious diseases has prompt researches on detection of pathogens [5,6]. Up to date, various methods have been developed and applied for detecting pathogens, including smear microscopy, isolation culture, biochemical reactions, and enzyme-linked immunosorbent assay (ELISA). However, these traditional detection methods suffer from drawbacks such as long detection time and complex operational steps, making them unsuitable for on-site pathogen detection at an early stage. With the development of molecular biology, nucleic acid-based pathogen detection methods have come to the forefront of infectious disease pathogen detection [7–9]. Especially, the polymerase chain reaction (PCR)-based nucleic acid detection method has become the gold standard in this field due to its high sensitivity, specificity, and accuracy [10–13].

Detection time is one of the important indicators for assessing the quality of a detection technology. The PCR process typically involves 3 main steps: denaturation (~93 °C), annealing (~60 °C), and extension (~70 °C), which means that it is a process that relies on thermal cycling [14–17]. However, the current mainstream PCR instruments still use thermoelectric coolers (TECs) as heating/cooling elements to heat the reaction tubes and their contents through metal baths [18,19]. Due to various unfavorable factors, such as the low power conversion efficiency of TECs (3% to 8%), the large volume of the metal bath, the low thermal conductivity of the reaction tubes, and the multi-layer heat conduction between different media, the thermal cycling time is too long to meet the demand for rapid detection. Aiming at accelerating the detection time, intensive investigation has focused on developing efficient heating mechanisms to replace the traditional Peltier-based heating schemes [20–22]. In recent years, the photothermal effect of nanomaterials has attracted extensive attention from researchers, which refers to the characteristic of materials converting light energy into heat energy under light irradiation, thereby rapidly increasing surface temperature [23–25]. Photothermal nanomaterials have been widely used in biomedical applications [26,27]. which also inspired the creative idea of developing a new generation of PCR heating technology. In 2015, the concept of ultrafast photonic PCR was proposed for the first time [28]. This technique uses photothermal nanomaterials as carriers and laser or other light as energy sources, achieving ultrafast thermal cycle regulation of the reaction system through photothermal heating, thereby enabling PCR amplification in a short time. Compared to traditional Peltier-based PCR, photonic PCR exhibits ultra-high photothermal conversion efficiency, ultra-fast heating rates, and ultra-low energy consumption, demonstrating enormous potential for the development of next-generation ultrafast PCR devices [28–31].

**Fig. 1.**
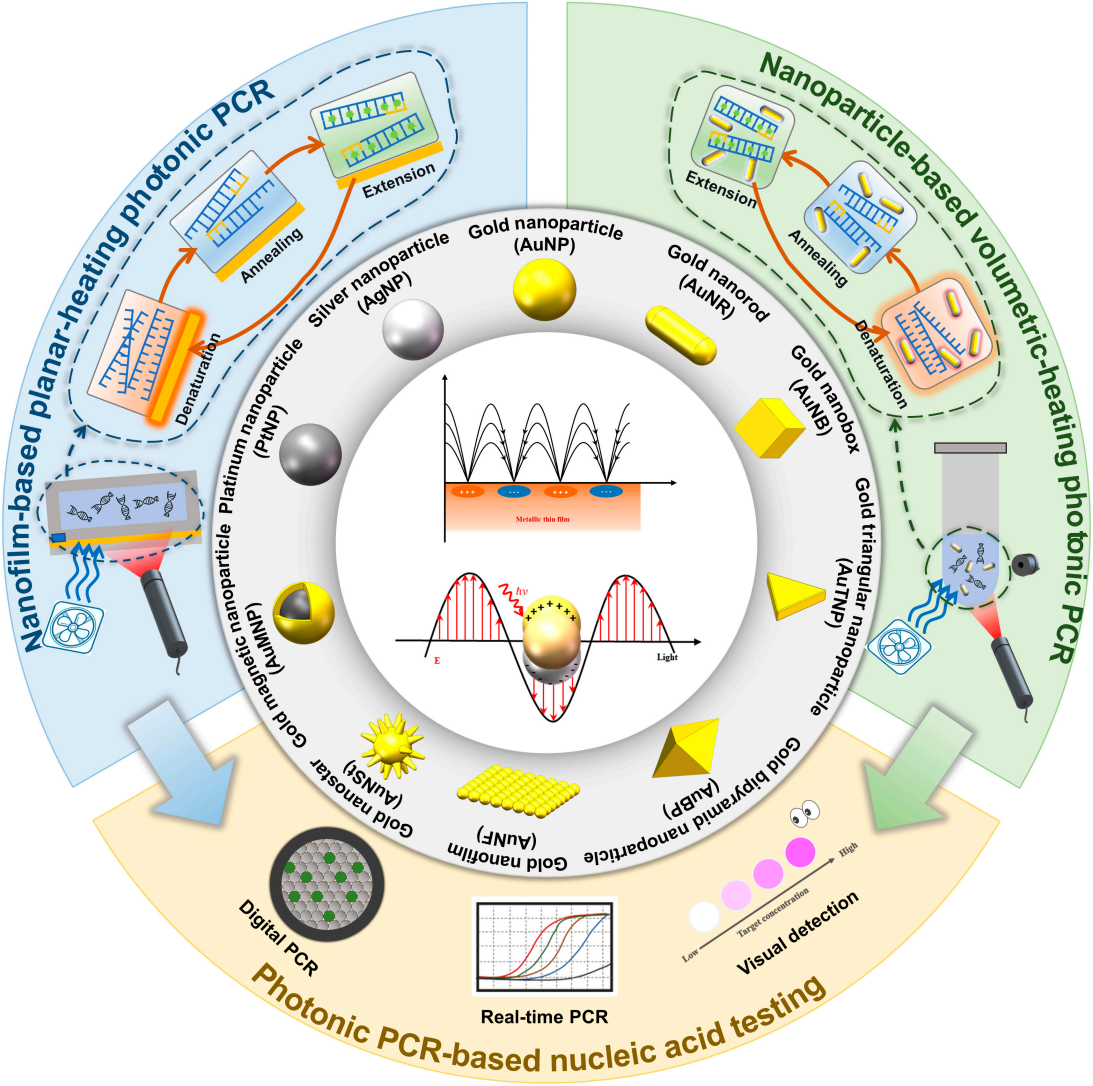
Schematic diagram of plasmonic heating principles, photothermal nanomaterials, planar-heating photonic PCR based on nanofilms and volumetric-heating photonic PCR based on nanoparticles, as well as photonic PCR-based nucleic acid testing.

This review comprehensively summarizes the latest advancements in photonic PCR, encompassing the photothermal effect mechanism, photothermal material structures, implementation methods and key technologies of photonic PCR, as well as the integration and applications of photonic PCR devices. As illustrated in Fig. 1, we first categorize various photothermal materials and elucidate their photothermal conversion mechanisms, including molecular thermal vibration, nonradiative relaxation, and plasmonic heating. Subsequently, based on the types of utilized photothermal materials and heating modalities, the photonic PCR techniques are divided into planar-heating photonic PCR, volumetric-heating photonic PCR, and other novel photonic PCR approaches distinct from these 2 heating paradigms. Representative research achievements for each type are detailed, along with a discussion of their advantages and limitations. Following this, the core technologies of photonic PCR are explored from 3 critical aspects: excitation light sources, temperature monitoring, and amplification product detection methods. Finally, we highlight 3 representative integrated nucleic acid detection systems based on photonic PCR and discuss their commercialization prospects in the field of nucleic acid point-of-care testing (POCT).

## Photothermal Effect Mechanisms

The photothermal effect refers to the physical phenomenon in which light energy is converted into heat energy after the interaction between light and matter [32,33]. This process can be summarized as follow: When a light beam irradiates the surface of materials, photons interact with the atoms, electrons, or molecules of the materials, causing part of the light energy to be absorbed and converted into the internal energy, leading to an increase in temperature of the materials [34]. Regarded as a representative phenomenon of light–matter interaction, the photothermal effect is widely present in various types of materials [35]. Different materials exhibit varying photothermal conversion capabilities and different conversion mechanisms depending on factors such as their number of free electrons or bandgap structure in response to electromagnetic radiation. In general, the photothermal conversion can be primarily categorized into plasmonic heating, molecular thermal vibration, and nonradiative relaxation [36,37]. In this section, we will attempt to elaborate on these 3 photothermal effect mechanisms in an easy-to-understand way.

### Plasmonic heating

The plasmonic heating effect typically occurs in noble metals such as gold (Au), silver (Ag), and platinum (Pt) [38,39]. Under the illumination of incident light, the free electrons on the surface of noble metal nanoparticles are excited by the incident light, resulting in collective oscillations. This will cause the local electron cloud to deviate from the atomic nucleus and create an asymmetric distribution on the particle surface. Simultaneously, under the influence of Coulomb force, the displaced electrons are attracted back to their original positions. Finally, subjected to the action of the above 2 forces, the electrons oscillate back and forth around their equilibrium position. This collective coherent oscillation of the conduction band electrons in some kinds of metal nanoparticles at the metal/dielectric interface is referred to as surface plasmons (SPs) [40]. SPs usually appear and propagate at the junction between metal nanoparticles (or nanofilms) and dielectric layers. When the frequency of the incident light aligns with the oscillation frequency of the electrons in the metal nanoparticles, a resonance phenomenon occurs, known as surface plasmon resonance (SPR) [41,42]. When metal nanoparticles undergo SPR, they strongly absorb photon energy and generate a high-intensity electric field on the particle surface [43].

**Fig. 2.**
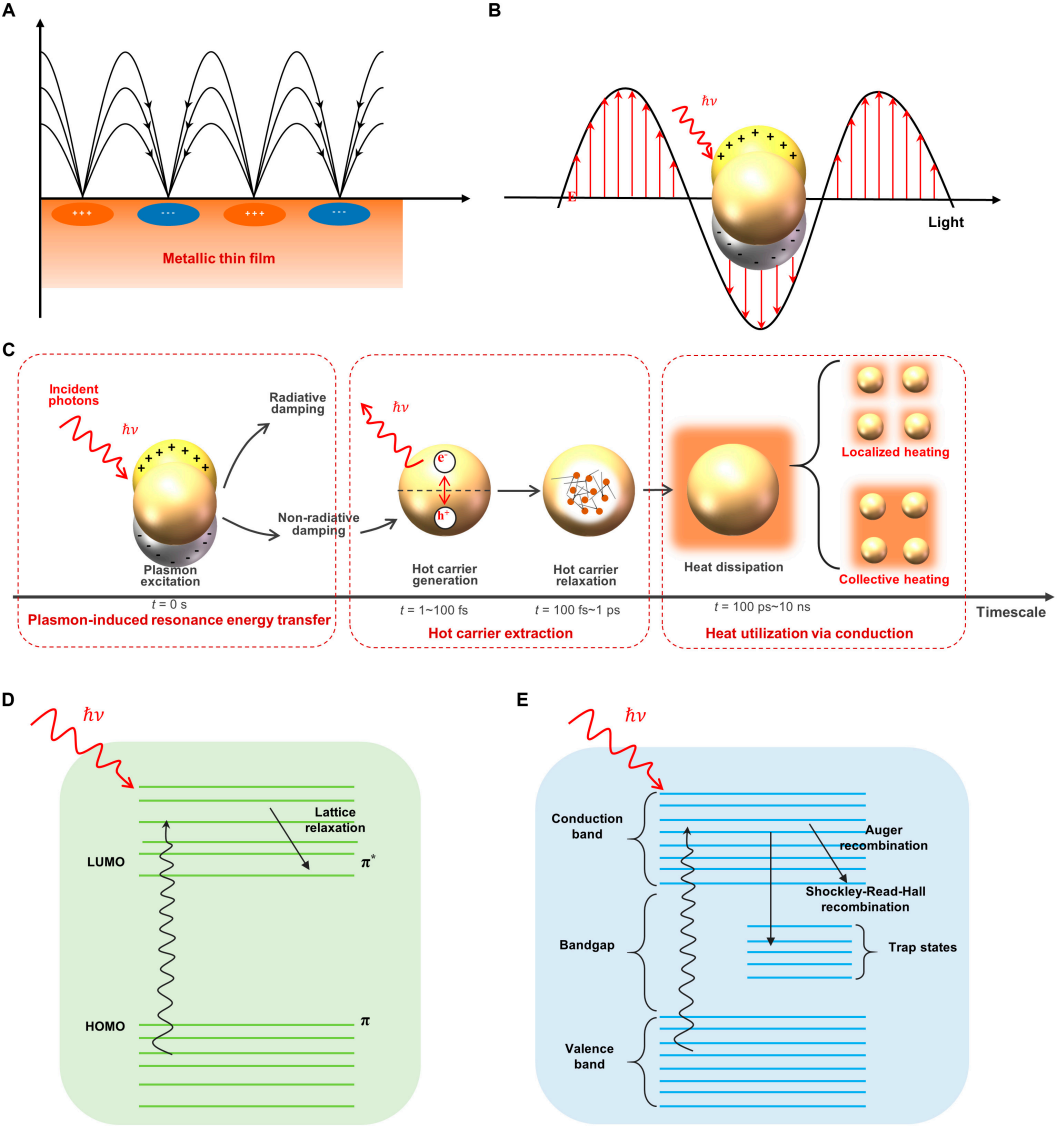
Schematic diagram of photothermal effect mechanisms. (A) SPP excited on a thin metal film. (B) Localized SP excited on a metal nanoparticle. (C) Localized SP and its characteristic timescales. Reprinted with permission from [36]. Copyright 2022, Wiley-VCH. (D) Molecular thermal vibration. (E) Nonradiative relaxation. Reprinted with permission from [37]. Copyright 2019, The Royal Society of Chemistry.

Generally speaking, SPR of metal nanomaterials can be mainly divided into 2 types. In the case of thin-film nanomaterials, when the wavelength of the incident light is shorter than the size of the nanofilm, it propagates along the film surface and oscillates at the dielectric interface, generating an oscillating electron cloud and thus producing heat (Fig. 2A), which is known as surface plasmon polariton (SPP) [36,44–46]. In another case, when the size of the nanoparticles is smaller than the wavelength of the incident light, the plasmonic nanostructures can break the diffraction limit of traditional optical devices, further confining the incident light within the nanoscale, thereby significantly enhancing the interaction between the incident light and the materials [47]. At this point, when the frequency of the incident light matches the oscillation frequency of the localized surface plasmons (LSPs) [48] of the metal nanoparticles, resonance takes place, resulting in strong absorption of light energy and the generation of a high-intensity local electric field near the particle surface (Fig. 2B). This local resonance phenomenon under specific incident light frequency is called localized surface plasmon resonance (LSPR). In LSPR, the amount of generated heat depends on the strength of the electron cloud’s oscillation, which is in turn primarily determined by on the resonance wavelength of the nanomaterials. Generally speaking, the resonance wavelength of a material is closely related to its geometric shape, size, and composition [49].

Figure 2C illustrates the process of SPP nanostructures absorbing photons and generating heat under illumination, depicted along a timeline [32,36]. The process can be divided into 3 steps: (a) Incident light irradiates the surface of metal nanoparticles, inducing plasmon resonance and exciting collective oscillations of surface electrons, where the local electric field can be enhanced by tens to hundreds of times, leading to strong absorption of the incident light energy [50]. (b) Within a time frame of 5 to 20 fs, SPs rapidly decay through radiative damping and nonradiative damping, exciting high-energy electrons within the structure. These high-energy electrons rapidly diffuse at Fermi velocity (approximately 10^6^ m/s) and redistribute their energy through electron–electron scattering processes, subsequently transferring energy to the lattice. (c) Within a time frame of 100 ps to 10 ns, the energy releases from the lattice to the surrounding medium in the form of thermal energy. It is noteworthy that if adjacent LSPs exhibit strong coupling, collective heating phenomena may occur, resulting in uniform heat distribution throughout the reaction system [51].

### Molecular thermal vibration

The photothermal effect, driven by light-induced molecular thermal vibrations, typically occurs in the organic semiconductors or those materials exhibiting organic semiconductor properties [33]. As illustrated in Fig. 2D, when the energy of the incident photons matches the energy required for electron transitions in the organic material, electrons absorb the photon energy and move from the ground state [highest occupied molecular orbital (HOMO)] to the excited state [lowest unoccupied molecular orbital (LUMO)] [52]. Subsequently, the electrons in the excited state will return to the ground state via 2 types of transitions: radiative transition and nonradiative transition. Among them, in the nonradiative transition, energy is released through electron–phonon coupling, leading to lattice vibrations and thus creating heat energy. It is noteworthy that the energy gap between HOMO and LUMO typically tends to decrease as the number of π bonds in the material increases, while the size of the energy gap between HOMO and LUMO determines the material’s capacity to absorbing photons. Specifically, the molecular photon absorption capacity increases as the energy gap decreases. On the other hand, the extent of molecular radiative transitions plays a crucial role in determining the photothermal conversion efficiency of the materials. As a result, carbon compounds, organic small molecules, and polymer materials with abundant conjugated π bonds are more likely to absorb photon energy, promoting the excitation of electrons from π orbitals to π* orbitals [53–55]. Subsequently, the absorbed energy is converted into thermal energy in the form of molecular thermal vibrations, exhibiting higher photothermal conversion efficiency [56].

### Nonradiative relaxation

The nonradiative relaxation photothermal effects typically occur in metal oxide semiconductors, referring to the process where electrons, excited by incident light, return to the ground state through interactions with the lattice, thus generating heat [57]. The nonradiative relaxation process usually involves electron–phonon coupling, where electrons transfer excess energy to the lattice, causing lattice vibrations and ultimately converting into heat. As depicted in Fig. 2E [33], for narrow bandgap semiconductor materials, the energy of the incident photons exceeds the bandgap energy, allowing electrons to transition from the valence band to the conduction band upon photon absorption, thus entering an excited state. Then, the electron–hole pairs generate above the bandgap by the electrons in the excited state. These electron–hole pairs reach the bandgap edge through Auger recombination and Shockley–Read–Hall recombination, transferring energy to the lattice, causing a local increase in lattice temperature, and ultimately leading to semiconductor heating [58], in which Auger recombination is a 3-carrier nonradiative process. When an electron and a hole recombine, the released energy is transferred to a third charge carrier (either an electron or a hole), promoting it to a higher-energy state within the same band. This high-energy carrier subsequently relaxes back to the band edge through multiple scattering events with lattice phonons, ultimately dissipating its excess energy as heat to the crystal lattice [59,60]. As for Shockley–Read–Hall recombination, this is a nonradiative recombination process mediated by defect states located within the semiconductor bandgap. A pair of opposite-type carriers are captured by the defect state, facilitating electron–hole pair annihilation. The energy released during recombination is transferred to the lattice primarily through lattice vibrations, leading directly to localized heating [61]. For semiconductor materials, the bandgap width directly determines their photothermal conversion efficiency. For semiconductors with wider bandgaps, on one hand, a portion of photons cannot be utilized because their energy is lower than the bandgap. On the other hand, the electron–hole pairs generated by photon excitation are closer to the bandgap edge, making them more likely to recombine and re-emit as photons, thereby reducing the photothermal conversion efficiency [62].

In summary, the 3 aforementioned photothermal effect mechanisms are all based on the interaction between photons and electrons in the material. Meanwhile, they exhibit different forms due to the different properties and internal structures of the materials. In metal materials, due to the existence of a large number of free electrons, thermal electrons are generated by inducing electron coherent oscillation and SPR through light irradiation, thereby releasing energy. In semiconductor materials, there are no large numbers of free electrons inside, and thermal energy is mainly released through nonradiative relaxation by electron–phonon coupling after the formation of electron–hole pairs above the bandgap through light irradiation. As for organic molecules and carbides, heat is released during the process of nonradiative transition of electrons from the excited state to the ground state accompanied by photon emission. In practical photonic PCR applications, there is no limitation for a single material or a single photothermal conversion mechanism. Composite materials composed of different photothermal materials have been prepared, and the comprehensive effect of the above mechanisms has further improved the photothermal conversion efficiency of the materials.

## Photothermal Nanomaterials and Ultrafast Photonic PCR

The widespread applications of photothermal nanomaterials in the field of biomedical such as drug delivery [63,64], disease treatment [65–69], sensing [70], and imaging [71] have inspired researchers to develop the new-generation ultrafast photonic PCR thermal cycling protocols. In recent years, various photothermal nanomaterials with different compositions and structures have been applied in the thermal cycling control of PCR, propelling the advancement of ultrafast photonic PCR technology [31,72,73]. Compared to those traditional thermocyclers that rely on Peltier devices for heating/cooling [21], the photonic thermal cyclers boast a higher power conversion efficiency, achieving faster heating rates with lower energy consumption. Moreover, the latter eliminates the requirement for the bulky radiator used for the nonworking face of the Peltier devices in traditional equipment [74,75], thus facilitating miniaturization of the device and applications in POCT field. In this section, we categorize the photonic PCR into planar-heating photonic PCR and volumetric-heating photonic PCR based on the heating modes and discuss each type separately. Under these 2 classifications, we summarize and categorize photonic PCR based on various photothermal nanomaterials. Furthermore, for planar-heating photonic PCR, we discuss the impact of factors such as material type, thickness, and surface structure on photothermal efficiency. For volumetric-heating photonic PCR, we compare the influence of factors such as nanoparticle type, shape, size, surface modification, and concentration on PCR.

### Nanofilm-based planar-heating photonic PCR

**Fig. 3.**
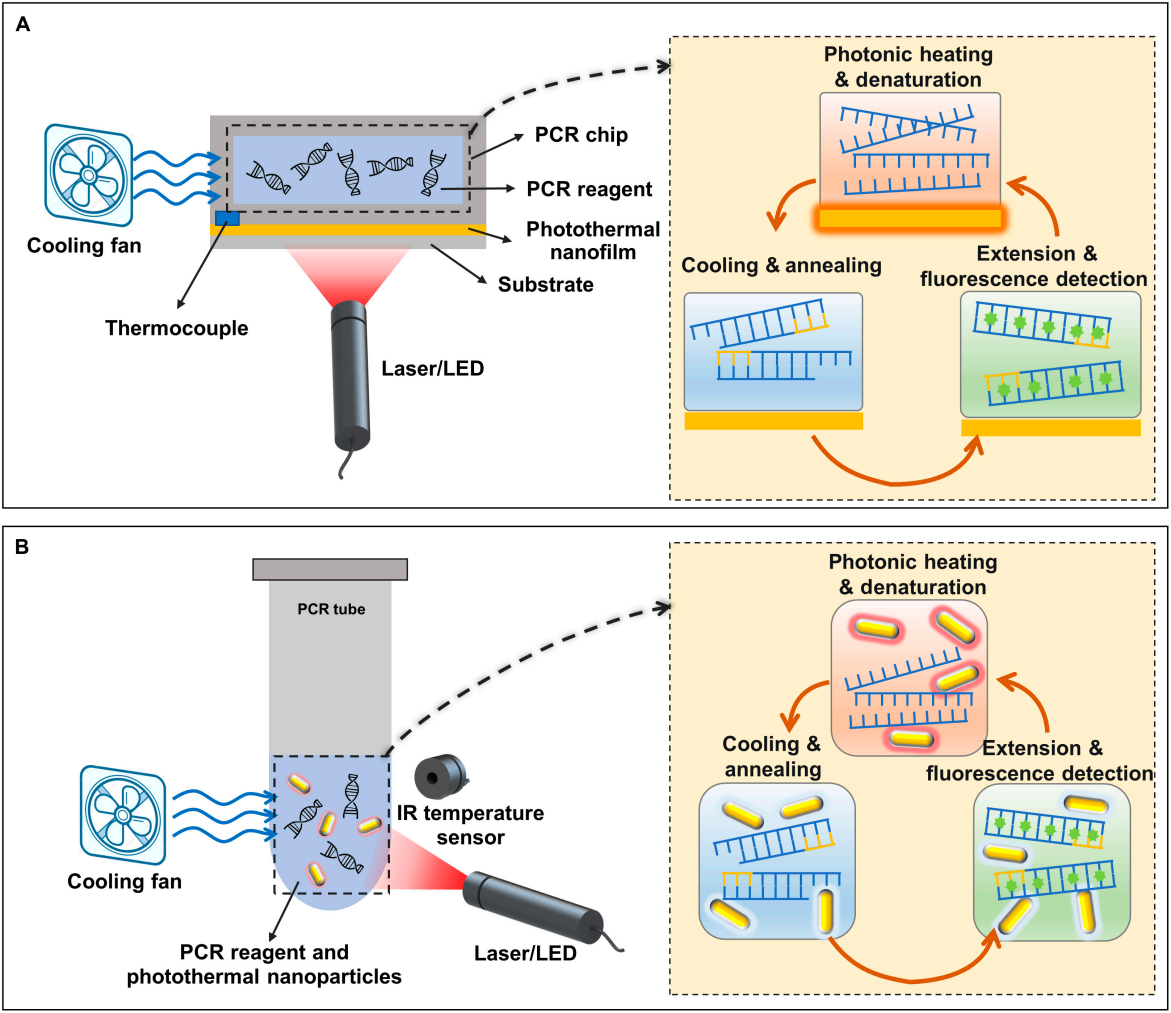
(A) Illustrative schematic of a typical planar-heating photonic PCR based on the SPP effect of nanofilms and (B) a typical volumetric-heating photonic PCR based on the LSPR effect of nanoparticles.

Inspired by the applications of membrane-based nanomaterials in photothermal therapy [76], distillation [77,78], antibacterial treatments [79], and so on, researchers have endeavored to apply them in the heating process of PCR thermal cycling. As shown in Fig. 3A, the working principle of the planar-heating photonic PCR is similar to that of traditional PCR. The difference lies in the replacement of the Peltier devices commonly used in traditional thermal cycler with a photothermal nanofilm, which can convert the energy of incident light into thermal energy and then heat the solution above it. Various types of photothermal nanofilms have been reported for PCR thermal cycling. Considering factors like photothermal conversion efficiency and thermal conductivity, the thickness of the nanofilms typically falls within the range of several tens to hundreds of nanometers. The nanofilms are usually fabricated on transparent substrates such as glass [80,81], polydimethylsiloxane (PDMS) [82,83], polymethyl methacrylate (PMMA) [84], and polycarbonate (PC) [85,86], using processes like sputtering or deposition.

Table 1 summarizes recent work on nanofilm-based planar-heating photonic PCR, encompassing aspects of nanofilm type and thickness, substrate material type, excitation source, heating/cooling rates, as well as thermal cycle time. By optimizing the film thickness, selecting excitation light sources with appropriate wavelengths and power, and reducing the volume of PCR solutions, the heating rate can be increased to over 10 °C/s [28,84], completing 30 PCR thermal cycles within 3.5 min [80]. In the following sections, we will provide a detailed introduction to these works.

**Table 1.** Recent plasmonic nanofilm-based planar-heating photonic PCR

Nanofilm type	Nanofilm thickness	Substrate material	Excitation source	Heating/cooling rates	Sample volume	Thermal cycle time	Reference
Au nanofilm	120 nm	PMMA	450-nm blue LED	12.79 ± 0.93 °C/s, 6.6 ± 0.93 °C/s	5 μl PCR mixture and 30 μl mineral oil	5 min (30 cycles from 55 to 95 °C)	[28]
Au nanofilm	120 ± 5 nm	PDMS	Two 450-nm blue LEDs	7.37 ± 0.27 °C/s, 1.91 ± 0.03 °C/s	20 μl water-in-oil droplets	13 min (30 cycles from 60 to 95 °C)	[83]
Au nanostructure	10 nm	Glass fiber membrane	785-nm laser	Heating to 95 °C in 4 s, cooling to 63 °C in 10 s	-	6 min (25 cycles between 63 and 95 °C)	[87]
N-heterocyclic carbene self-assembled monolayer-based Au film	160 nm	PDMS	447-nm blue LED	8.75 °C/s, 17.5 °C/s	-	40 cycles in under 8 min	[88]
Au nanofilm	160 nm	Glass wafer	447 nm LED	4.40 ± 0.18 °C/s, 8.86 ± 0.15 °C/s	-	8 min (40 cycles between 60 and 94 °C)	[97]
Two gold nanofilms	20 nm + 100 nm	PMMA	50-W high-power white LED	13.20 °C/s, 7.92 °C/s	20 μl	7.5 min (30 thermal cycles)	[84]
Gold nanoislands	10–50 nm	Glass nanopillar arrays	White LED	9.3 °C/s, 12.4 °C/s	15 μl	3.5 min (30 cycles between 98 and 60 °C)	[80]
Chrome layer and gold layer	5 nm + 115 nm	PDMS/PMMA	Four bule LED	3.3 ± 0.2 °C/s, 8.0 ± 0.4 °C/s	-	50 min (10-min hot start with 40 cycles of PCR)	[82]
Carbon-black film	14 μm	PET	Near-IR (940 nm) LED	22 °C/s 2.6 °C/s	5 μl	7 min (30 thermal cycles of the standard PCR)	[114]
Au, TiO_2_, and Bi layer	100 nm + 100 nm + 150 nm +90 nm	PET	808-nm laser	-	30 μl	A PCR amplification cycle in ~2.5 min	[210]
Gold dendritic nanoforests (Au DNFs)	~3.5 μm	Si	250-W halogen lamp	-	25 μl PCR reagent and 200 μl mineral oil	45 s (PCR cycles between 60 and 97 °C)	[106]
Ti and Au	5 nm + 80 nm	PC	Two 447.5 nm blue LEDs	-	20 μl	10 min (40 cycles between 95 and 62 °C)	[86]
Au film	120 nm	PMMA	3 W blue LED	7.50 ± 0.46 °C/s, 6.35 ± 0.49 °C/s	1.3–10 μl	4 min (1.3 μl, 30 thermal cycles between 94 and 68 °C)	[253]

#### Photonic PCR based on Au nanofilms

**Fig. 4.**
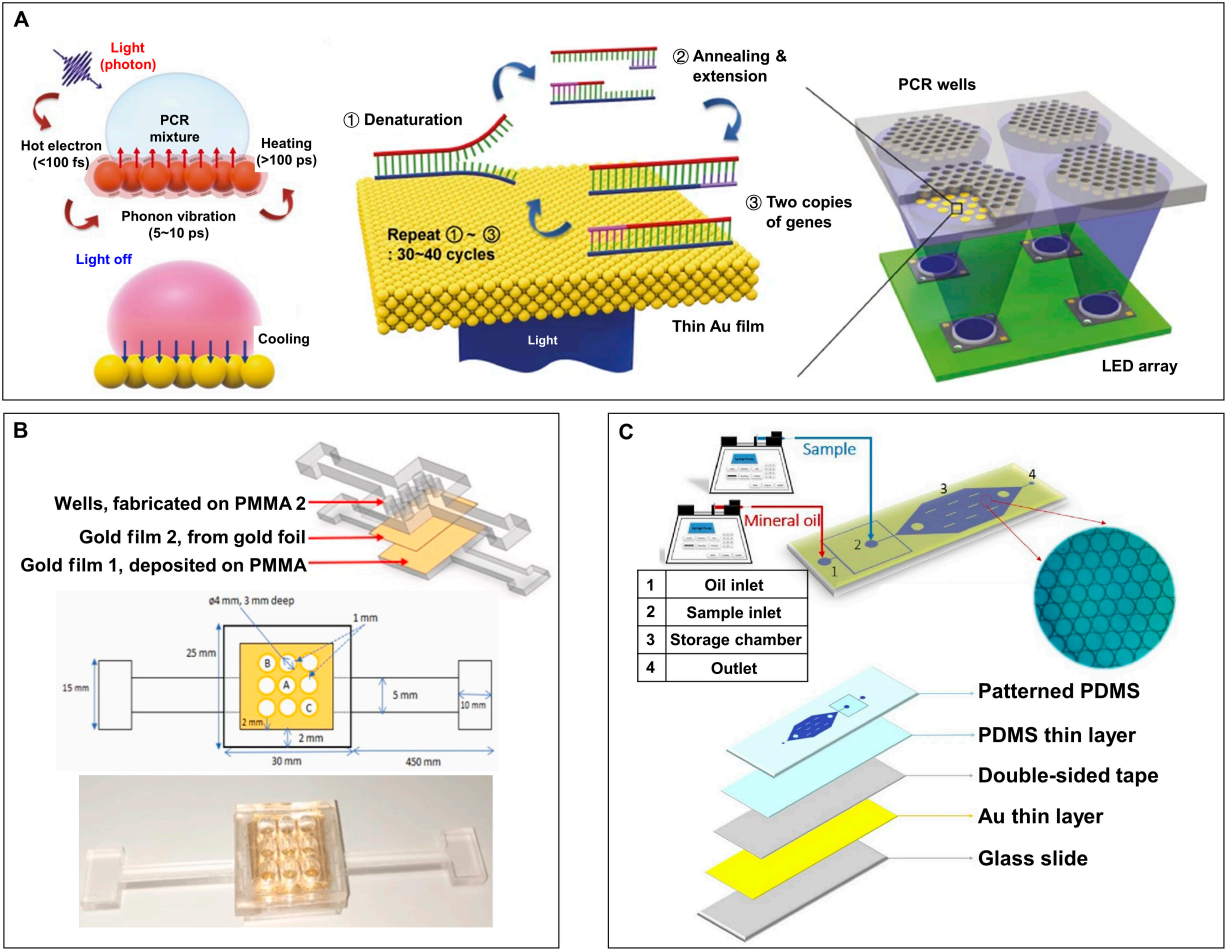
AuNF-based planar-heating photonic PCR devices. (A) Schematic illustration of the working principle and working flow of photonic PCR on the AuNFs illuminated by LEDs. Reproduced with permission from [28]. Copyright 2015, The authors, published by Springer Nature. (B) Schematic diagram (top) and photograph (bottom) of the plasmonic chip with double-layer gold films. Reproduced with permission from [84]. Copyright 2023, The authors, published by Elsevier. (C) Schematic diagram of the plasmonic photothermal microfluidic dPCR chip and its multi-layer sturcutre. Reprinted with permission from [83]. Copyright 2021, The authors, published by Springer Nature.

Because of its exceptional photothermal conversion efficiency, gold (Au) nanofilms (AuNFs) are the most commonly used materials in planar-heating photonic PCR [28,83,87,88]. One feasible approach involves assembling the AuNFs onto a transparent substrate via methods such as sputtering and deposition, thereby constructing a photothermal PCR microfluidic chip. Then, heating of the liquid within the microfluidic chip can be achieved by irradiating the nanofilm with excitation light of a specific wavelength. In 2015, Lee and colleagues [28] initiated research on photonic PCR with thin gold films as the heat source. They cut a series of 4-mm-diameter cylindrical holes on a PMMA sheet with thickness of 4 mm, used another PMMA sheet with thickness of 1.5 mm as the base plate, and thermally bonded them together to form PCR wells. Then, the AuNFs of varying thicknesses (10 to 120 nm) were deposited onto the base plate using electron beam evaporation to construct planar-heating photonic PCR wells (Fig. 4A). A blue light light-emitting diode (LED) was used as the excitation light source to conduct 30 thermal cycles on the solution (5 μl of reaction liquid with 30 μl of mineral oil) with an ultrafast heating (12.79 ± 0.93 °C/s) and cooling (6.6 ± 0.29 °C/s) rate, successfully achieving the amplification of λ-DNA in 5 min. In another work, Nabuti and colleagues [84] developed a photonic PCR chip featuring double-layer AuNFs to further enhance the heating and cooling rates, while also minimizing temperature deviation to improve the uniformity of reagent temperature during thermocycling. As shown in Fig. 4B, they adhered 100-nm gold film 2 (prepared by assembling a 5-nm-thick pure gold foil) on 20-nm gold film 1 (prepared by depositing on a PMMA substrate via radio frequency magnetron sputtering) to serve as the base of the PCR wells for plasmonic heating. Because of the excellent photothermal conversion efficiency and thermal conductivity of the double-layer AuNFs, the heating and cooling rates of the constructed photonic PCR chip can reach 13.2 and 7.92 °C/s, respectively.

The advancement of microfluidic technology and microdroplet generation techniques has facilitated the emergence of a revolutionary molecular biology technology—digital PCR (dPCR) [89,90]. Unlike real-time PCR, dPCR minimizes the impact of competition between targets and enables absolute quantification detection without the need for standard curves, significantly improve the sensitivity of the detection [91–93]. In recent years, dPCR has become an invaluable tool for applications like viral analysis, copy number variations, and gene expression analysis [94–96]. In order to further improve the detection speed and simplify the instrument design, some researches have reported the combination of the plasmonic photothermal effect with dPCR microfluidic chips [97–100]. Jalili et al. [83] have developed a plasmonic photothermal dPCR device based on AuNFs. As shown in Fig. 4C, the microfluidic chip made of PDMS was fabricated using conventional photolithography techniques and comprised a multi-layer structure. From bottom to top, the layers include a glass substrate, Au film, transparent double-sided tape, a PDMS thin layer, and a patterned PDMS. A 120-nm-thick AuNF was deposited on the surface of the glass substrate by direct current magnetron sputtering. The AuNF was irradiated with a peak wavelength of 450-nm LED to heat the liquid in the PDMS chip chambers above it. To further enhance the photothermal heating rate, 2 excitation LEDs were placed both beneath and atop the AuNF. The system demonstrated mean heating and cooling velocities of 7.37 °C/s (SD ±0.27) and 1.91 °C/s (SD ±0.03), respectively, accomplishing 30 thermal cycles between 60 and 95 °C within 13 min.

#### Photonic PCR based on AuNFs with novel structure

**Fig. 5.**
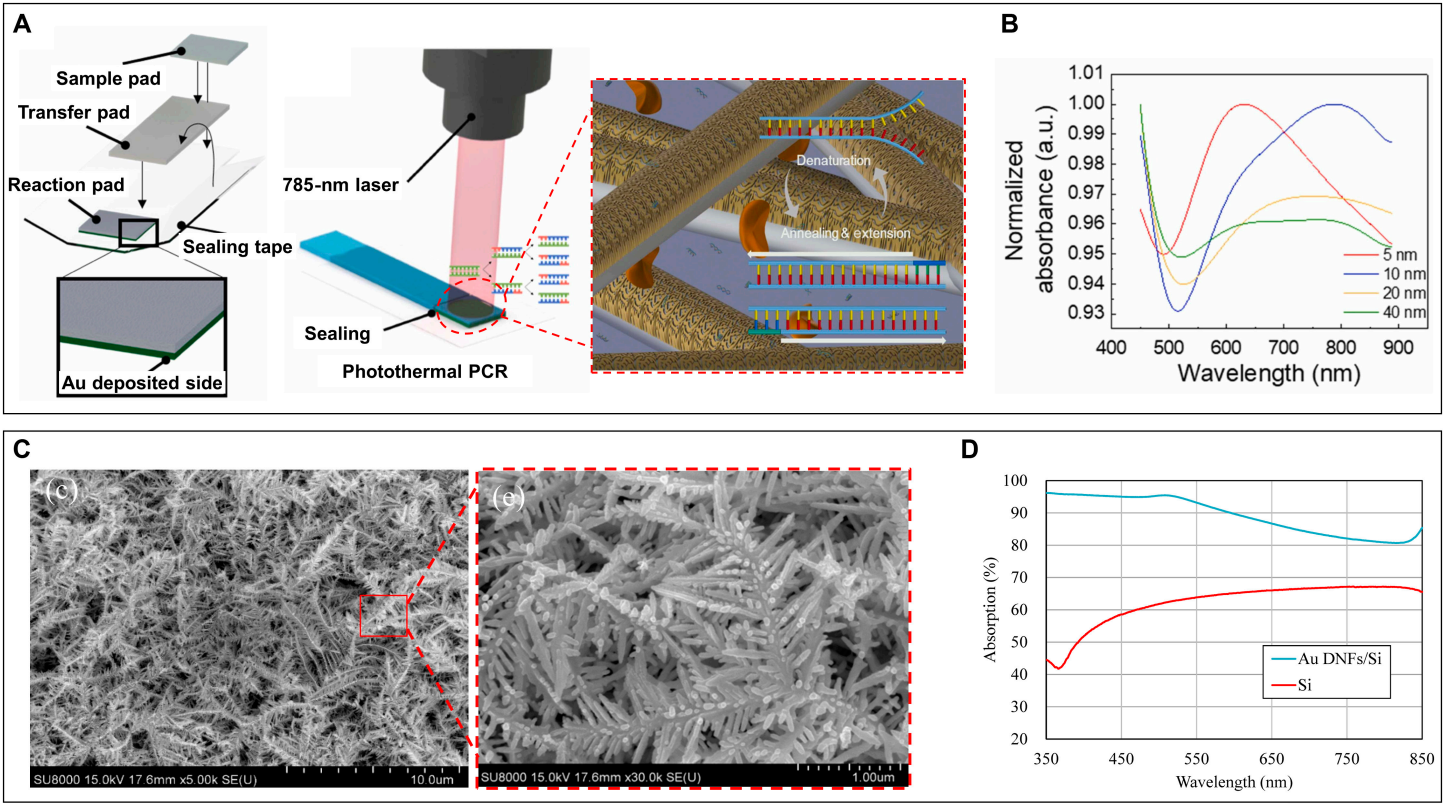
AuNFs with different thickness and AuNFs based on differnet substrate material. (A) Schematic diagram illustrating the structure of the photonic PCR platform based on the glass fiber membrane-based AuNF (left) and photonic PCR process on this glass fiber membrane-based AuNF (right). (B) The absorption spectra of films with different Au deposition thicknesses show significant variations in absorption peaks and intensity as the thickness changes. Reproduced with permission from [87]. Copyright 2022, Elsevier. (C) Scanning electron microscopy (SEM) photographs of the Au DNF/Si chip. (D) Absorption spectra of the Au DNF/Si chip and the pure Si chip. The absorption rate of Au DNF/Si chip is significantly higher than that of the pure Si chips in the visible light range. Reproduced with permission from [106]. Copyright 2020, The authors, published by MDPI.

The thickness variation of AuNFs significantly affects their absorption of excitation light energy at different wavelengths, thereby influencing the photothermal conversion efficiency [101,102]. Moreover, the type of substrate material also impacts the films’ absorption of photon energy [103–105]. Kim and colleagues [87] deposited a thin Au film on glass fiber membranes using electron beam evaporation to form a photonic PCR chip. As shown in Fig. 5A, the glass fiber membrane consists of numerous intertwined glass fibers, creating a substantial internal space. Consequently, gold ions can permeate into these spaces and form Au nanostructures on both the surface and interior of the membrane, which increases the surface area of the Au nanostructures, thereby enhancing the photothermal conversion efficiency. By depositing a 10-nm-thick Au nanostructure on glass fiber membranes and using a 785-nm laser for radiative heating, a minuscule amount of reagent absorbed by the membrane can be heated to 95 °C within just 4 s. They carried out 25 thermal cycles between 63 and 95 °C on the reagent absorbed by the membrane within 6 min and accomplished the amplification and analysis of *Staphylococcus aureus* totally within 12 min by detecting the fluorescent signal of SYBR dye. They also deposited Au with thicknesses ranging from 5 to 40 nm on the films to investigate the absorption intensity of Au nanostructures at different wavelengths. As indicated in Fig. 5B, the Au nanostructures with thicknesses of 5 and 10 nm developed a green coloration and exhibited broad absorption near the 600- and 800-nm bands, respectively. When the Au deposition thickness exceeded 20 nm, the color turned gold, indicating the formation of an Au film, and the absorption spectrum range expanded. Therefore, we can conclude that achieving optimal photothermal conversion efficiency requires matching the appropriate thickness of AuNFs with the suitable excitation wavelength. Furthermore, fabricating AuNFs incorporating elements of different structures and sizes could enable strong light absorption across multiple wavelength bands simultaneously, thereby enhancing the photothermal conversion efficiency. In another research, in order to obtain a plasmonic nanofilm with broadband-light absorption capacity, Lin and colleagues [106] synthesized Au dendritic nanoforests (Au DNFs) on the silicon (Si) chips to form Au DNF/Si chip for PCR (Fig. 5C). Figure 5D shows that the chip has high absorption rate in the visible wavelength range compared to the pure Si chip. By utilizing a standard halogen lamp as the photonic heating source, DNA fragments of *Salmonella spp.* were successfully amplificated and detected on this photonic PCR chip system.

#### Photonic PCR based on carbon-black films

**Fig. 6.**
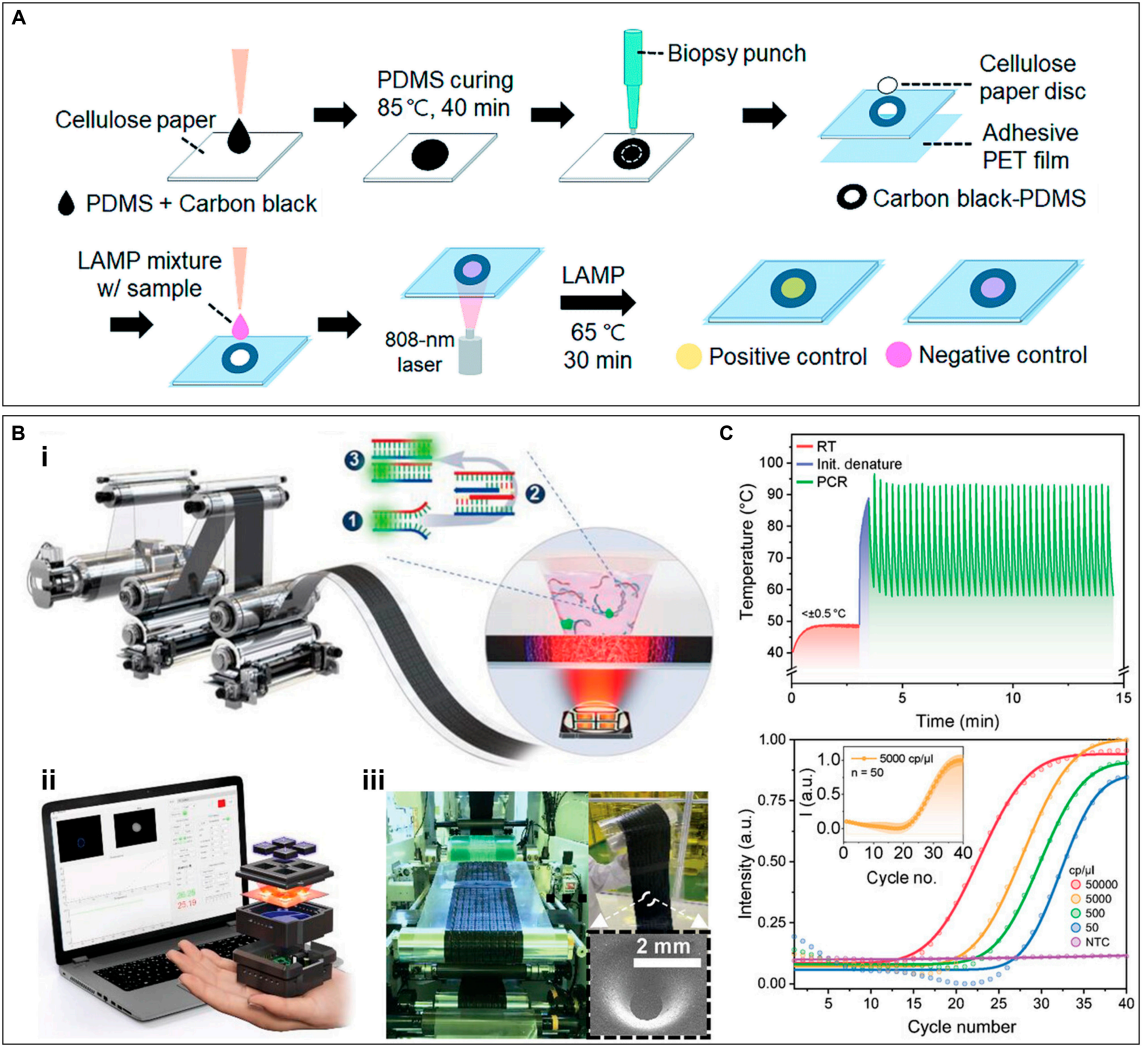
Carbon black-based planar-heating photonic PCR devices. (A) Fabrication and working principles of the paper-based chip patterned with carbon black–PDMS film. Reproduced with permission from [109]. Copyright 2022, The Royal Society of Chemistry. (B) MEDIC-PCR platform. (i) Schematic diagram of the roll-to-roll (R2R) gravure and imprinter for print carbon-black thin film and the process of photonic PCR based on the film. (ii) Portable and compact MEDIC-PCR device and its supporting software running on the laptop. (iii) Large-scale, fully automated manufacturing of PCR chip array using R2R gravure printed and imprinted technologies. (C) Thermal cycle curves (above) and fluorescence intensity curves (below) of RT-qPCR carried out on the MEDIC-PCR device. Reprinted with permission from [114]. Copyright 2023, The authors, published by Wiley-VCH

The noble metals like Au and Pt are mostly expensive and difficult to process into nanofilms, making the cost of AuNF-based disposable chips too high to be adopted for wide use [107,108]. Therefore, researchers have tried to use other photothermal materials for membrane-based photonic PCR platform. For instance, Joong’s team [109] developed a paper-based chip patterned with a carbon-black and PDMS mixture for photonic nucleic acid amplification. Figure 6A illustrates the fabrication steps and working principle of the paper-based photonic heating device, where the photothermal region is a ring composed of a mixture of PDMS and carbon black. Taking advantage of the photothermal characteristics of carbon black, a 3-mm-diameter area in the center of the ring was heated to 65 °C under laser irradiation with a wavelength of 808 nm. Loop-mediated isothermal amplification (LAMP) was used to amplify the foodborne pathogen *Escherichia coli* O157:H7 with results determined by visual observation of color change within 15 min. Compared with traditional heating schemes, this method has lower power consumption and faster heating speed. In addition, the target temperature can be easily adjusted by altering the laser power or the concentration of the carbon-black film, which also provides imagination for the use of this device for photonic PCR.

The large-scale batch manufacturing method can not only reduce the cost of the testing consumables but also improve the yield rate and reduce the difference between batches, which is the only way for all testing consumables or devices to move from the laboratory to clinical application [110–113]. As shown in Fig. 6B, another study also reported a rapid mobile efficient diagnostics of infectious diseases via on-chip reverse transcriptase quantitative PCR (MEDIC-PCR) based on the carbon-black (multi-layered graphene and amorphous carbon hybrid) thin film [114]. In particular, in order to reduce the time and money consumption of chip fabrication, they developed a roll-to-roll gravure printing technology to print different thicknesses of carbon-black film on the polyethylene terephthalate (PET) films, with a flexographic plate as molds to imprint polymer-based microwells, enabling the large-scale, fully automated manufacturing of disposable PCR chips. Based on the fabricated photothermal PCR chip, the authors reported a portable palm-sized point-of-care (POC) device to enable mobile and efficient diagnosis of infectious diseases. The device integrates 4 near-infrared (NIR) LEDs with an optical power of 3.75 W, which can operate 4 PCRs simultaneously. As shown in Fig. 6C, under NIR light irradiation, the carbon-black film induces electron–phonon and phonon–phonon couplings, thereby facilitating the photothermal conversion process and reaching a heating rate of up to 24 °C/s. This device is capable of delivering sample to answer of nucleic acid within 15 min while maintaining comparable accuracy and sensitivity to conventional real-time PCR systems.

The heating implementation of planar-heating photonic PCR is similar to traditional conductive heating methods based on TECs and metal thermal wells. It can heat the reagents directly (the heated reagent is contacted with the nanofilm directly) or indirectly (with a protective layer between the nanofilm and the heated reagent) to regulate PCR thermal cycling. However, compared to traditional PCR instruments, planar-heating photonic PCR has lower power consumption and higher heating efficiency and is more conducive to miniaturization of devices. Certainly, there are some challenges that currently limit the further clinical application of planar-heating photonic PCR. Firstly, although direct heating of the reagents by the nanofilms can effectively improve heating efficiency and shorten thermal cycling time, the inhibitory effect of the nanofilms on PCR can reduce amplification efficiency, thus affecting detection sensitivity [115–117]. Secondly, as indicated in Table 1, in most studies, AuNFs excited and heated by blue light at a wavelength of around 450 nm were selected as the photothermal films. However, the bule light is always used as the excitation light for many PCR fluorescent dyes, thereby affecting fluorescence detection of PCR. Additionally, the expensive cost and complex deposition processes involved in nanofilms based on noble metals also serve as key factors limiting the further widespread application of planar-heating photon PCR. However, most of the current researches focus only on portion of the aforementioned issues and has not proposed a perfect solution. In the future, a comprehensive approach should be considered, including material selection, process optimization, consumable structure improvement, and methodological innovation, paving the way for the practical application of planar-heating photon PCR.

### Nanoparticle-based volumetric-heating photonic PCR

Figure 3B illustrates working principles of the volumetric-heating photonic PCR based on photothermal nanoparticles. Different from the planar-heating photonic PCR, nanoparticles are directly added into the PCR reagents and uniformly suspended in the solutions. Then, under the irradiation of excitation light, each nanoparticle acts as a tiny heating unit, directly heating the surrounding solution [118]. Thus, compared with the planar-heating photonic PCR, this approach can significantly enhance the heating rate [119]. Roche et al. [120] reported a real-time plasmonic quantitative PCR (qPCR) based on Au nanorods (AuNRs), which can deliver a 30-cycle PCR in 54 s. Besides, Richardson et al. [121] have found that when the nanoparticle concentration is high enough, the light-to-heat power conversion efficiency can reach almost 100%. Metal nanoparticles undergo plasmon resonance under light irradiation, which can produce high temperatures of more than 1,000 °C/s [122–124]. Building on this, surface modification and coating of metal nanoparticles can enhance their thermal stability and reduce their impact on biological reactions [125–127]. Moreover, by incorporating a magnetic core, various composite nanomaterials with both photothermal effects and superparamagnetism can be developed, presenting exciting application prospects [99,128,129]. Table 2 lists the details of the applications of various nanoparticles in volumetric-heating photonic PCR.

**Table 2.** Recent plasmonic nanoparticle-based volumetric-heating photonic PCR

Nanoparticle type	Nanoparticle characteristic	Surface modification	NP concentration	Excitation source	Heating/cooling rates	Sample volume	Thermal cycle time	Ref.
AuNP	Diameter: 60 nm	PEG-8000	4.4–17.9 pM	2.7-W 532-nm laser	7.62 ± 0.81 °C/s, 3.33 ± 0.24 °C/s	25 μl + 150 μl oil	~700 s (30 cycles between 45 and 90 °C)	[127]
AuNR	Diameter: 10 nm Length: 41 nm	PEGylate with HSPEG5000	10–50 nM	2-W 808-nm laser	72 °C/s, 50 °C/s	25 μl + 30 μl oil	54 s (30 cycles)	[120]
Magnetic Fe_3_O_4_ nanocluster	Cube length: 10–11 nm	-	2,500 ppm	460-mW 808-nm laser	4.5 °C/s, 2.25 °C/s (calculated)	10 μl	420 s (30 cycles between 55 and 95 °C)	[175]
AuNR	Aspect ratio of 4	mPEG-thiol	0.24 nM	5.6-W 808-nm laser	6.25 °C/s, 1.99 °C/s (calculated)	10 μl + 3 μl oil	19 min (30 cycles between 40 and 85 °C)	[125]
Au bi-pyramid (AuBP)	PEG-Si-AuBPs	Silica shell coat, mPEG-silane-5000	OD 0.6–34.0	850-nm IR-LED	16.6 ± 2.4 °C/s, 9.4 ± 0.8 °C/s	5 μl + 15 μl wax	141.8 ± 12.4 s (30 cycles between 72 and 95 °C)	[168]
Magneto plasmonic nanoparticle	Core: Zn_0.4_Fe_2.6_O_4_ Core diameter: 16 nm Shell: Au Shell thickness: 12 nm	Phosphine sulfonate ligands	OD 4	Six 80-mW 532-nm laser	13.17 °C/s, 4.94 °C/s	10 μl	11 min of RT-PCR by magneto-plasmonic thermocycling	[128]
Au/Ag triangular	-	-	0.2–1.9 mM	1.29 W 808-nm laser	-	1 μl	-	[169]
Magneto plasmonic nanoparticle	Core: SiO_2_ Au Fe_2_O_3_ Shell: Au Overall diameter: 150 nm	Silica coat and mPEG-silane-10000	OD 12.30	8.5-W 850-nm IR-LED	7.69 ± 0.23 °C/s, 5.89 ± 0.15 °C/s	10 + 20 μl oil	250 s (30 cycles between 72 and 95 °C)	[234]
AuNR	-	Functionalized silica coat	OD 18	Three 850-nm IR-LED	6.7 ± 0.2 °C/s, 4.7 ± 0.1 °C/s	10 + 75 μl oil	<15 min (contains 50 °C for 2 min, 95 °C for 10 s, and 45 cycles between 60 and 95 °C)	[156]
Magnetic graphene oxide	-	Agarose microcarriers	-	20-W 808-nm laser	8.2 °C/s, -	127,000 droplets with diameter of 72 μm (195 pl)	-	[99]
AuNR	-	Gelatin	OD 12.80	1 W/cm^2^ 980-nm laser	30.5 °C/s, 9.8 °C/s	10 μl	~2.5 min (30 cycles between 65 and 95 °C)	[126]

Among various metal nanoparticles, Au nanoparticles (AuNPs) play a crucial role in biomedical applications [130–135], such as bioimaging [136], biosensing [137,138], cancer therapy [139], drug delivery [140], hyperthermic therapy [141], and antibacterial activity [142], due to their multifunctional surface chemistry, unique optoelectronic properties, and excellent biocompatibility [143,144]. Additionally, in the field of molecular biology, several studies have demonstrated that the addition of an appropriate amount of AuNPs to PCR reagents can enhance both the yield and specificity of PCR amplification [145–149]. This may be due to the fact that AuNPs can selectively bind to the single-stranded DNA (ssDNA), which greatly reduces the mismatch between primers and templates during DNA replication [150,151]. In another study, Li et al. [152] significantly enhanced the sensitivity of PCR and reduced the reaction time by introducing 0.7 nM AuNPs with diameter of 13 nm into the PCR system. The addition of AuNPs to the conventional PCR system increased detection sensitivity by 5 to 10 times, while in the real-time PCR system, the sensitivity was improved by more than 10^4^ times. In conclusion, the primary mechanisms by which AuNPs enhance PCR sensitivity can be summarized as follows: (a) The high thermal conductivity of AuNPs accelerates heat transfer within the reaction system, promoting both the denaturation of double-stranded DNA templates and primer-template annealing, thereby improving amplification efficiency [153]. (b) AuNPs can selectively adsorb ssDNA, reducing primer mismatch and inhibiting nonspecific amplification, thus enhancing specificity. However, this adsorption is highly concentration-dependent; excessive AuNPs inhibit the reaction by adsorbing DNA polymerase or template DNA [150]. (c) The LSPR effect of AuNPs can enhance the signal intensity of nearby fluorophores, significantly lowering the detection limit in real-time PCR [154,155]. However, there are also other studies that have shown that nanoparticles, such as gold nanorods (AuNRs), have a fluorescence quenching effect that can affect the detection results [128,156]. In contrast to AuNPs, anisotropic AuNRs exhibit dual effects: Fluorescence enhancement can occur if the longitudinal plasmon band matches the emission wavelength, whereas mismatched transverse modes may cause quenching. Importantly, this quenching effect is strongly dependent on particle shape and size. For instance, a related study has found that AuNRs can quench dopamine fluorescence through the inner-filter effect [157]. Furthermore, the properties of the fluorophore also influence the outcome; fluorophores with low intrinsic quantum yield tend to benefit most from plasmonic enhancement, while stable fluorophores can undergo competitive quenching if the spacing is suboptimal. This apparent paradox is not a fundamental conflict [158]. By optimizing parameters such as nanoparticle concentration, spacer distance, nanoparticle geometry, and environmental conditions, it is possible to selectively achieve either fluorescence enhancement or quenching.

**Fig. 7.**
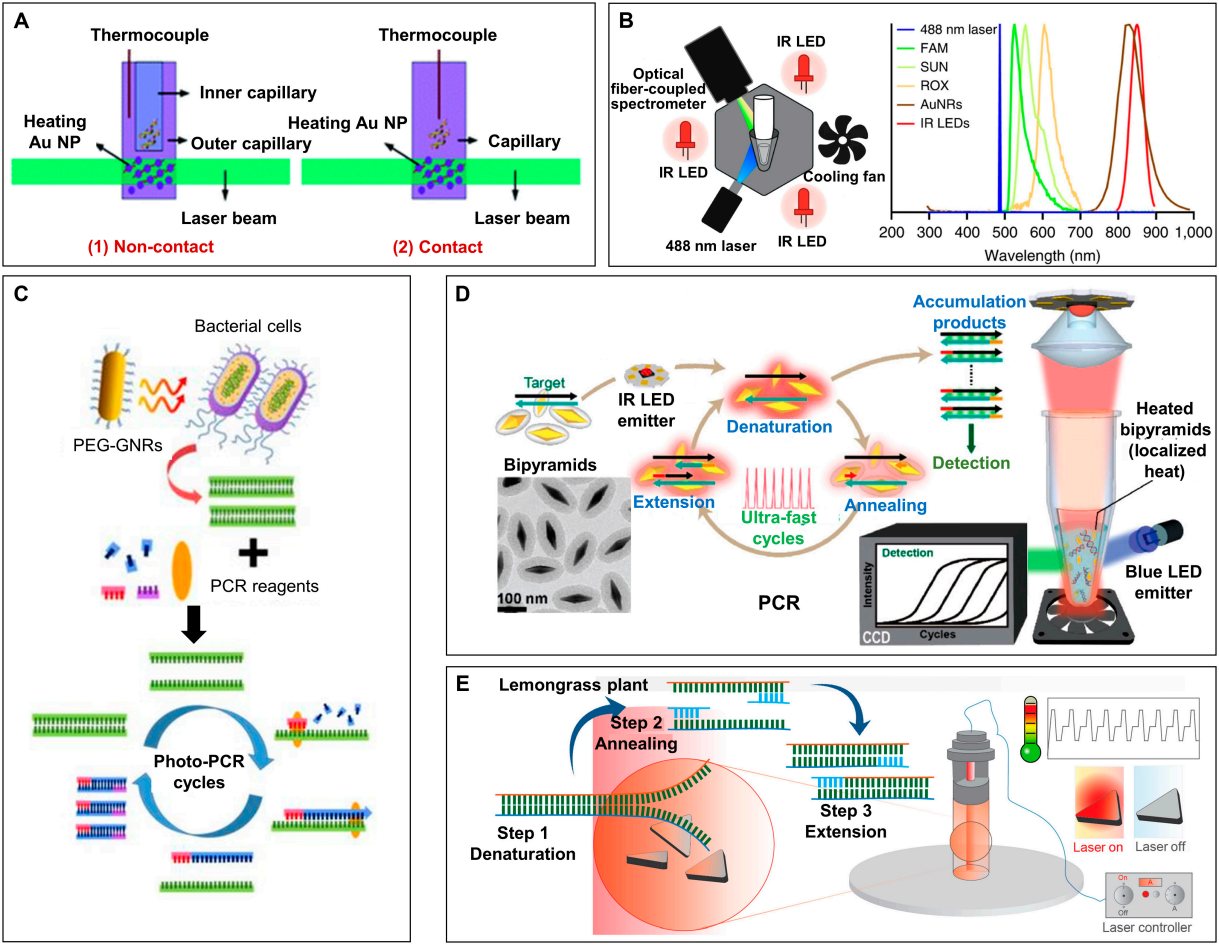
Volumetric-heating photonic PCR based on gold nanoparticles. (A) Comparative schematic diagram of noncontact (left) and contact (right) photonic PCR methods based on AuNPs. Reproduced with permission from [127]. Copyright 2012, The Royal Society of Chemistry. (B) Schematic illustration of the photonic real-time RT-PCR instrument, in which 3 IR LED is used for plasmonic heating, a cooling fan is used for cooling, and a 488-nm laser as well as a spectrophotometer are used for fluorescence detection (left); graph illustrating the nonoverlapping optical spectra of common fluorescence detection and IR LED excitation (right). Reprinted with permission from [156]. Copyright 2022, The authors, published by Springer Nature. (C) Schematic illustration of one-step DNA extraction and photonic PCR of bacterial cells based on AuNRs. Reprinted with permission from [125]. Copyright 2017, Ivyspring International Publisher. (D) TEM image of the synthesized PEGylated and silica-coated gold bipyramid nanoparticles (PEG-Si-AuBPs), plasmonic photothermal (PPT)-based nucleic acid amplification schemes, and scheme of the LED-assisted PPT device based on PEG-Si-AuBPs. Reprinted with permission from [168]. Copyright 2017, American Chemical Society. (E) Schematic illustration of the machine-free PCR based on the plasmonic photothermal effect of AuTNPs and AgTNPs. Reprinted with permission from [169]. Copyright 2020, American Chemical Society.

In 2012, Roche’s team [127] first applied AuNPs to PCR thermal cycling control. As shown in Fig. 7A, they examined 2 heating modes of contact and noncontact, and the results showed that contact heating can significantly reduce the thermal cycle time (the time required for 30 thermal cycles has decreased from 45 min to 10 min). They also investigated the inhibitory effect of different concentrations of AuNPs on polymerase in contact heating mode and proposed the use of additives such as bovine serum protein to reduce the potential inhibitory effect of AuNPs on PCRs. Since then, the volumetric-heating photonic PCR based on different kinds of plasmonic nanomaterials has been demonstrated among the numerous studies.

Compared to AuNPs, AuNRs exhibit a higher photothermal conversion efficiency [159–162] and are more frequently utilized in photothermal PCR cycles. A significant advantage of AuNRs is that their plasmonic resonance wavelength within the NIR spectral window can be precisely regulated by altering their aspect ratio, achieving optimal matching with the excitation light source, thus enhancing the photothermal conversion efficiency [163,164]. Additionally, the plasmonic resonance band of AuNRs lies within the NIR range (~800 nm), which does not overlap with the excitation and emission spectra of commonly used fluorescent probes and dyes in PCR, thereby allowing real-time in situ fluorescence excitation and detection without removing the AuNRs (Fig. 7B, right). Based on this, as shown in Fig. 7B (left), Sia and colleagues [156] developed a photonic reverse transcription PCR (RT-PCR) system with real-time in situ fluorescence monitoring. An optical device composed of 3 infrared (IR) LEDs operating at 850 nm was utilized to rapidly heat a 20-μl solution within thin-walled PCR tubes. Because of the efficient absorption of IR LED energy by AuNRs, this system attained a heating rate of 6.7 ± 0.2 °C/s and a cooling rate of −4.7 ± 0.1 °C/s, which help the system to complete the RT-PCR amplification in 15 min, which included 2 min for reverse transcription and 45 thermal cycles. In another study, Jon’s team [125] utilized poly(ethylene glycol)-modified gold nanorods (PEG-GNRs) as a photothermal heat source for both cell lysis and PCR thermal cycling. As illustrated in Fig. 7C, by irradiating with an 808-nm laser, they integrated bacterial cell lysis and DNA amplification into a single step. Research has shown that modifying the surface of AuNRs with PEG can significantly minimize the electrostatic interactions between AuNRs and PCR reagents, and improve aqueous dispersity [165,166]. In addition, another study proposed gelatin-AuNRs, which enhanced the thermal stability and biocompatibility of the AuNRs without compromising their photothermal conversion efficiency. Experiments have shown that even after 40 thermal cycles, the gelatin-AuNRs maintained their original morphology and good dispersibility [126].

In photothermal PCR, uniform and even heating of the entire solution is crucial for achieving amplification homogeneity. To achieve uniform heating, nanoparticles need to exhibit characteristics such as minimal polydispersity in both shape and size, a narrow absorbance bandwidth, and a high extinction coefficient [167]. As shown in Fig. 7D, Weizmann and colleagues [168] proposed gold bipyramid nanoparticles (AuBPs) featuring highly tunable longitudinal SPR and low polydispersity (<2%), which exhibit a higher absorbance coefficient compared to nanospheres and nanorods. Using IR LEDs as the excitation light source, the thermal cycling of PEG-Si-AuBPs at different light densities was measured, with a 21.5 optical density (OD) sample having 30-cycle time of 141.8 ± 12.4 s, corresponding to significant heating and cooling rates of 16.6 ± 2.4 °C/s and 9.4 ± 0.8 °C/s, respectively. Maji’s team [169] synthesized triangular gold nanoparticles (AuTNPs) and triangular silver nanoparticles (AgTNPs) and attempted to perform a low-cost, machine-free PCR based on an 808-nm NIR laser (Fig. 7E). They also found that compared to nanospheres, calf-thymus DNA (ct-DNA) binds significantly to AuTNPs and AgTNPs, but there are differences between the 2, where AgTNP stabilizes ct-DNA, while AuTNP disrupts the stability of DNA molecules.

**Fig. 8.**
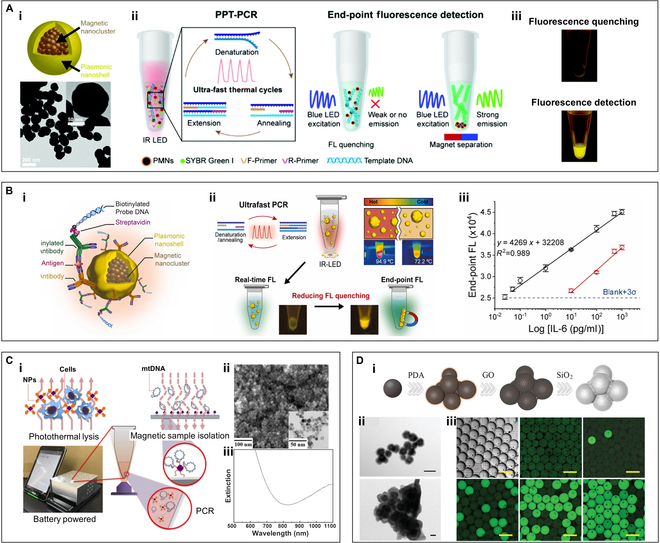
Volumetric-heating photonic PCR based on composite nanoparticles. (A) PPT-PCR method based on the PMNs. (i) Scheme and TEM image of the bifunctional PMNs. (ii) Schematic diagram of the PPT-PCR method with in situ end-point fluorescence detection. (iii) Fluorescence photographs of the amplification products before (above) and after (below) magnetic separation. Reproduced with permission from [129]. Copyright 2021, The Royal Society of Chemistry. (B) PIMNs and the PPT-qiPCR detection method for IL-6 quantification. (i) Schematic structure of the PIMN. (ii) Schematic illustration of PPT-qiPCR with dual readout of real-time and end-point fluorescence. (iii) End-point fluorescence curves of the same sample before (red plots) and after (black plots) the magnetic separation. Reprinted with permission from [176]. Copyright 2023, Elsevier. (C) Magneto-optical qPCR device based on dual-mode magnetic Fe_3_O_4_ NCs. (i) Schematic depiction of the magnetic field-driven nucleic acid manipulation and ultrafast photonic PCR on a handheld battery-powered system. (ii) SEM image of the Fe_3_O_4_ NCs. (iii) NIR absorption profile of the Fe_3_O_4_ NCs determined by spectrometry. Reprinted with permission from [175]. Copyright 2016, The authors, published by Springer Nature. (D) NIR-responsive ddPCR based on magnetic GO composite. (i) Synthesis flowchart of the Fe_3_O_4_@GO@SiO_2_ nanocomposite. (ii) TEM images of Fe_3_O_4_@GO (above) and Fe_3_O_4_@GO@SiO_2_ (below). (iii) Bright-field image of the nanocomposite (the first one) and the fluorescence image of the nanocomposite with different concentrations of DNA spike after amplification. Reprinted with permission from [99]. Copyright 2022, Wiley-VCH.

In the aforementioned study, various metal nanoparticles, represented by gold nanoparticles, have been extensively applied in photonic PCR, demonstrating significant advantages over traditional PCR thermal cyclers in terms of reaction time, equipment miniaturization, and energy consumption. However, owing to the severe fluorescence quenching effect by AuNPs, most AuNP-based photonic PCRs require extra post-processing and separate result reading steps, making in situ real-time fluorescence detection challenging [170,171]. This process will undoubtedly increase the overall processing time of photonic PCR-based nucleic acid testing, diminishing the benefits of rapid photothermal cycling. Consequently, researchers attempted to develop composite photothermal nanomaterials to address the last shortcoming of photonic PCR. Among these, magnetic nanoparticles (MNPs) are given priority due to their efficient magnetic field response characteristics [128,172–174]. In 2016, Shieh and colleagues [175] first reported novel dual-mode magnetic Fe_3_O_4_ nanoclusters (NCs) that provide photothermal conversion properties. Utilizing these Fe_3_O_4_ NCs, they successfully developed a highly efficient nucleic acid molecular manipulation based on magnetic field control as well as ultrafast photonic real-time PCR method for the detection of *Clostridium difficile* infections (Fig. 8C). By controlling the size of the synthesized Fe_3_O_4_ NCs, they retained the thermal stability and magnetic responsiveness of Fe_3_O_4_ while overcoming the limitation of the common nonstoichiometric structure of Fe_3_O_4_, which made it difficult to obtain photothermal properties in the NIR wavelength region. As depicted in Fig. 8C (iii), the Fe_3_O_4_ NCs exhibit a V-shaped optical absorption curve at 700 and ~1,000 nm.

Constructing composite magnetic photothermal particles using an MNP core and a photothermal shell is considered as another viable approach, in which gold is the most common photothermal shell material due to its efficient photothermal conversion efficiency [128,129,174,176,177]. For instance, Lee’s team [129] reported an in situ endpoint fluorescence detection plasmonic photothermal PCR (PPT-PCR) based on core–shell plasmonic magnetic nanoparticles (PMNs). To achieve enhanced saturation magnetization and photothermal properties, as illustrated in Fig. 8A (i), the PMNs were composed of a gold shell and a superparamagnetic NC core, which consisted of large numbers of small-sized iron oxide nanoparticles. Figure 8A (ii) shows the schematic principle of the PPT-PCR, where PMNs were used to achieve 30 thermal cycles between 72 and 95 °C within a 10-μl reaction system in 4.2 min. Subsequently, magnetic separation was employed to remove the PMNs, reducing fluorescence quenching and enabling high-sensitivity in situ fluorescence signal detection. Figure 8A (iii) displays the fluorescence intensity of the amplification products before and after magnetic separation, demonstrating that removing PMNs via magnetic separation significantly enhances the fluorescence intensity. Furthermore, based on the aforementioned PMNs, the team developed a multifunctional plasmonic immunomagnetic nanoparticles (PIMNs) and constructed a plasmonic photothermal quantitative immuno-PCR (PPT-qiPCR) detection method for accurate and swift quantification of interleukin-6 (IL-6) through dual readout of real-time and endpoint fluorescence (Fig. 8B) [176]. Figure 8B (i) shows the schematic structure of the PIMNs, which were labeled with anti-human IL-6 monoclonal capture antibody and biotinylated anti-human IL-6 monoclonal detection antibody on the PMN base, detecting human IL-6 through a sandwich immunoassay. The biotinylated detection antibody pre-assembled with streptavidin–DNA template served as a signal probe in the PCR. During PCR, the fluorescence signal of the indicator probe DNA was monitored in each cycle. Besides, at the endpoint of the PCR, PIMNs were removed from the solution using simple magnetic separation (which takes 15 s), and the in situ end-point fluorescence signal of the amplicon was detected, enabling quantitative detection of IL-6 (Fig. 8B, ii). The entire analysis could be completed within 10 min. In Fig. 8B (iii), the red and black segments show the endpoint fluorescence signals of the same sample before and after magnetic separation, respectively. The detection sensitivity and dynamic range of the in situ endpoint fluorescence detection can be significantly enhanced due to the elimination of fluorescence quenching effect of the nanoparticles through magnetic separation.

In addition to gold, it has also been reported that other photothermal materials are used as photothermal shells to construct composite photothermal materials. As shown in Fig. 8D, Ye and colleagues [99] constructed a core–shell composite (Fe_3_O_4_@GO@SiO_2_) by sequentially encapsulating graphene oxide (GO) and silica around a magnetic nanocore (Fig. 8D, i) and embedded this composite into agarose microcarriers to obtain a NIR-responsive composite agarose. Figure 8D (ii) shows the transmission electron microscopy (TEM) images of Fe_3_O_4_@GO and Fe_3_O_4_@GO@SiO_2_. Using a flow-focusing microfluidic device, they emulsified a pre-mixture of the nanocomposite, agarose, and PCR solution to generate doped agarose microcarriers. This innovative approach led to the development of a new NIR-responsive thermal cycling method for droplet digital PCR (ddPCR), enabling the detection of *Klebsiella pneumoniae* DNA over a wide concentration range (Fig. 8D, iii). In another study, they selected MXene (Ti_3_C_2_T*_x_*, where T represents O, OH, or F groups) as a photothermal shell and synthesized a Fe_3_O_4_@MXene@SiO_2_ composite using a similar method [178]. Based on this, they developed a novel exosome-derived miRNA analysis strategy using the photothermal ddPCR method for the diagnosis of prostate cancer and other applications.

In the volumetric-heating photonic PCR, the photothermal nanoparticles, which serve as the heating units, are directly added to the PCR reagents and uniformly distributed. This allows the heat to be directly transferred from the nanoparticles to the reagents, exhibiting an enhanced heating rate compared to the planar-heating scheme. On the other hand, the addition of photothermal nanoparticles also increases the complexity of the PCR system. Issues such as the inhibition of the amplification reaction by the nanoparticles, fluorescence quenching by the nanoparticles [179,180], and the stability of the nanoparticles during thermal cycling require further investigation [181]. The focus of research lies on the modification and enhancement of photothermal nanoparticles to improve their performance in certain aspects. For example, surface coating and modification can endow nanoparticles with higher thermal stability and specific adsorption functions. Additionally, the development of composite photothermal nanoparticles by combining magnetic cores with plasmonic shells enables magnetic separation and manipulation through an external magnetic field.

### Other novel forms of photonic PCR

The 2 sections above introduced 2 of the most typical technical routes of photonic PCR: the nanofilm-based planar-heating method and the nanoparticle-based volumetric-heating method. For the nanofilm-based photonic PCR, on the one hand, the selectivity of the material types is limited, and on the other hand, the thickness of the nanofilm deposition needs to be finely controlled to ensure efficient absorption of excitation light energy [182–184]. Most importantly, the spectral range of photothermal excitation light of the AuNFs overlaps with the spectral range of the excitation light of common fluorescence dyes and probes used in real-time fluorescence PCR, which restricts the application of fluorescence detection in PCR. As for nanoparticle-based photonic PCR, although numerous studies have increased the photothermal conversion efficiency of nanoparticles by changing their shape, size, and surface modification [185–188], the aggregation of nanoparticles during thermal cycling, the inhibition of PCR, and the quenching effect on fluorescence remain challenges. In this section, we will introduce several new attempts that are different from the above 2 technical routes as a complement to photonic PCR technology.

**Fig. 9.**
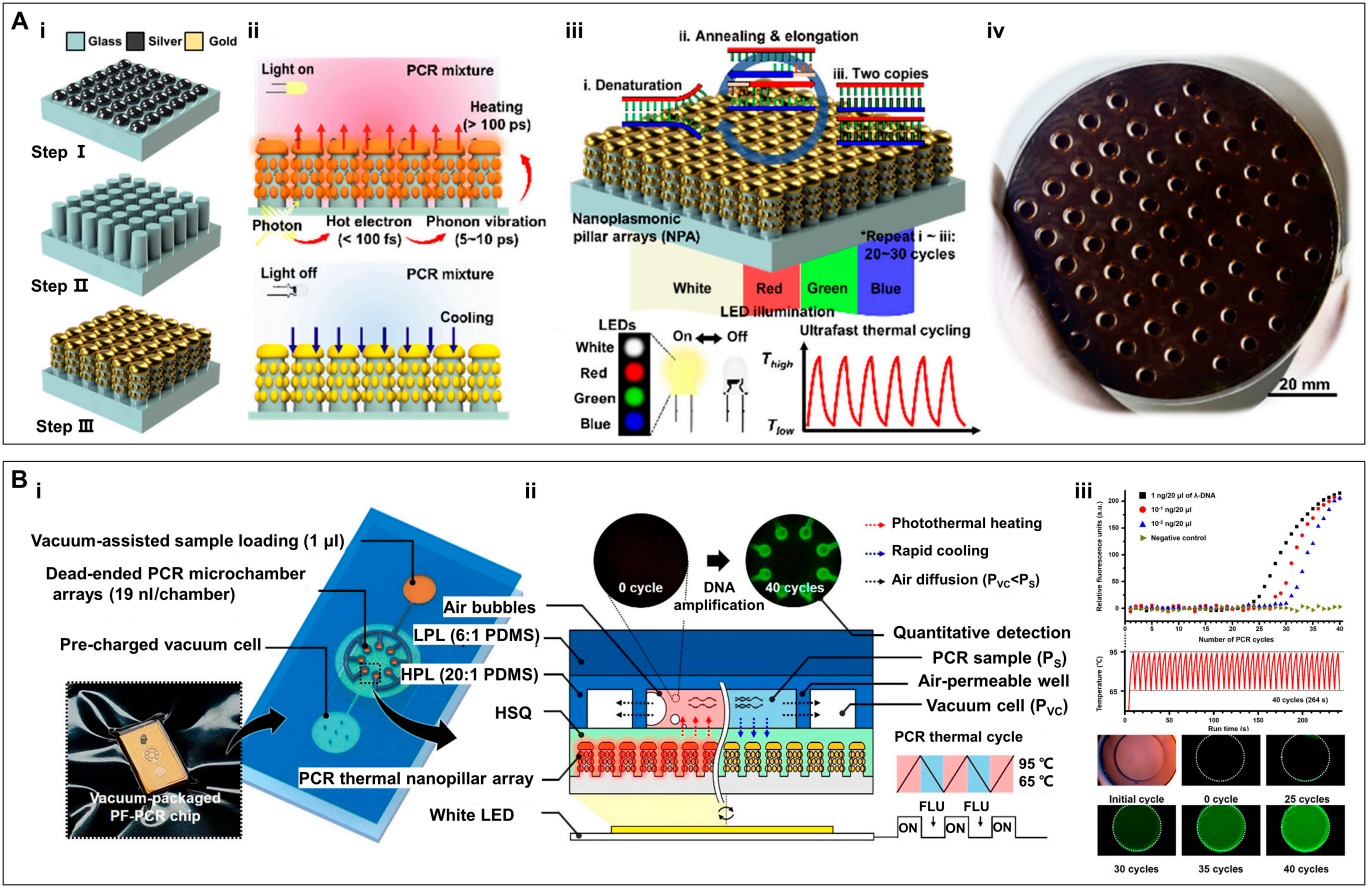
The NPA and the vacuum-charged PF-PCR chip based on the NPA. (A) (i) Fabrication steps of the NPA. (ii) Schematic diagram of the photothermal light to heat conversion on the NPA. (iii) Schematic illustration of the ultrafast nanoplasmonic PCR based on the NPA under the white LED illumination. (iv) Photograph of a wafer level NPA substrate with PDMS multiwell arrays. Reproduced with permission from [80]. Copyright 2020, American Chemical Society. (B) (i) Schematic diagram of the PF-PCR chip. (ii) Schematic working principle of the vacuum-assisted nanoplasmonic on-chip PCR. (iii) Real-time PCR curves (top), thermal cycles curves (middle), and fluoresncece image (bottom) of the amplification of λ-DNA by the on-chip PCR. Reproduced with permission from [81]. Copyright 2021, The authors, published by American Chemical Society.

The optimal absorption wavelength of a plasmonic nanomaterial is closely related to its size; thus, a plasmonic nanomaterial of specific sizes generally has a fixed single absorption peak [185]. Therefore, a viable method to enhance the photothermal conversion efficiency is to construct size variations and 3-dimensional distributions of nanogaps, enabling the materials to have a high absorption rate across the entire visible light spectrum. As illustrated in Fig. 9A (i), Jeong’s team [80] fabricated glass nanoplasmonic pillar arrays (NPAs) with Au nanoislands (AuNIs) on a bare silicon wafer through 3 steps: (a) fabricating Ag nanoislands on a borosilicate glass substrate using the solid-state dewetting method; (b) forming nanopillar arrays on the glass substrate through reactive ion etching technology; (c) depositing AuNIs onto both the top and sidewall of the formed glass nanopillars using thermal evaporation. The AuNIs on the NPA have various sizes and gaps, capable of converting the full spectrum of visible light into thermal energy, thereby significantly enhancing the utilization rate of the excitation light (Fig. 9A, ii). Using a high-power white light LED as the excitation light source, the NPA completes 30 thermal cycles between 60 °C/s and 98 °C in 3.5 min, achieving average heating and cooling rates of 9.3 and 12.4 °C/s, respectively (Fig. 9A, iii). Subsequently, as shown in Fig. 9B (i and ii), combining the NPA with the microfluidic channels fabricated through photolithography, replica molding, and oxygen plasma treatment, they constructed the vacuum-assisted plasmofluidic PCR (PF-PCR) chip. Using a white LED as the excitation light source, the surface of NPA with nanopillars 180 nm in height can reach 100 °C in 8 s and attain a saturation temperature of 200 °C. Figure 9B (iii) shows the amplification and quantification of λ-DNA on the PF-PCR chip. This system can complete 40 cycles of λ-DNA amplification in 264 s and 40 cycles of severe acute respiratory syndrome coronavirus 2 (SARS-CoV-2) amplification in 306 s [81]. This work creatively constructed a NPA containing AuNIs of different sizes to 3-dimensionalize the 2-dimensional nanofilm through abundant gaps between the nanopillars and different sizes of AuNIs to achieve highly enhanced light absorption throughout the entire visible range. The results show that under identical white LED illumination, the NPA exhibited a strong light energy absorption of 2.3 times greater than the general AuNFs.

**Fig. 10.**
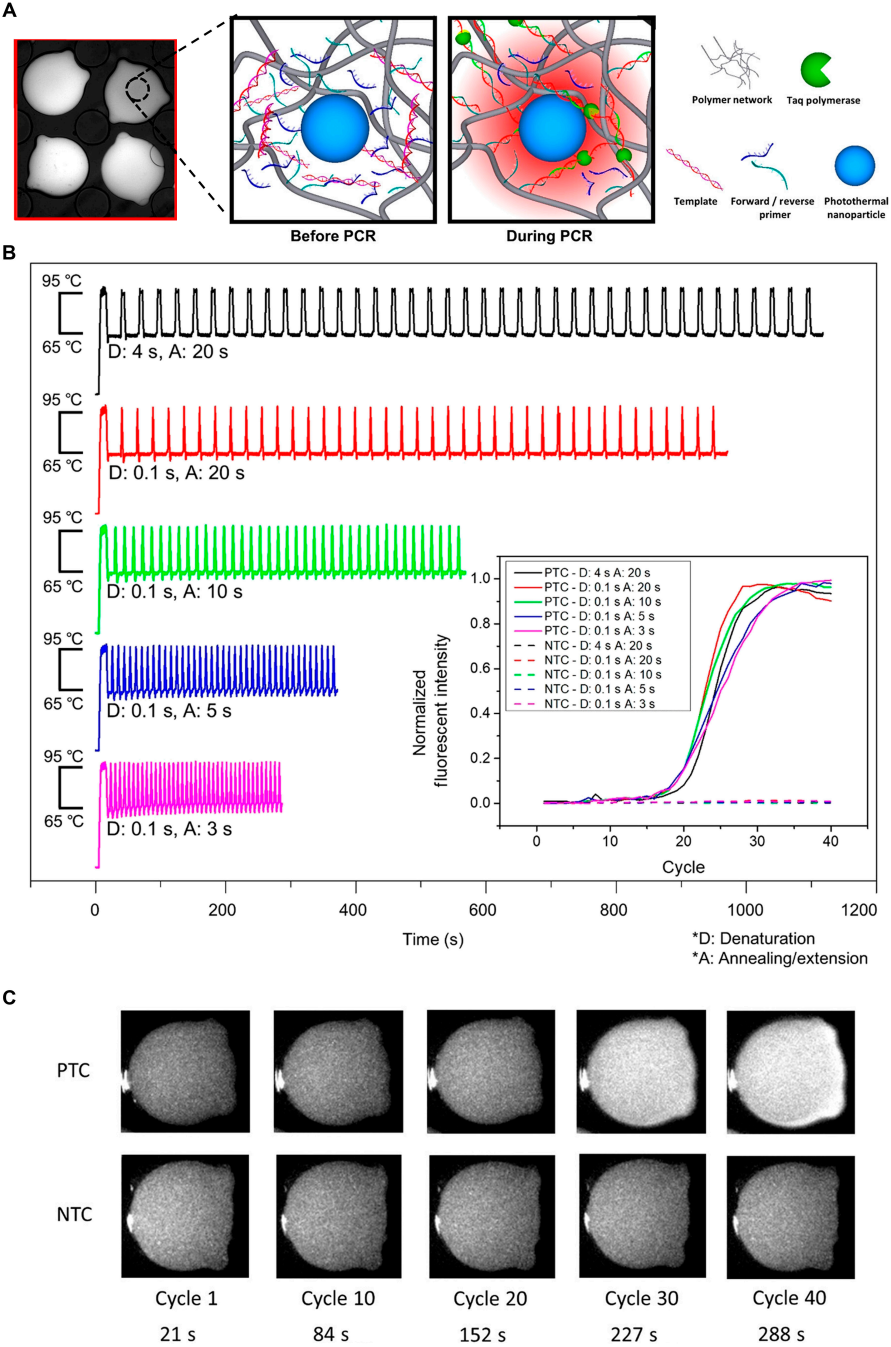
Ultrafast real-time PCR in the pPINs. (A) Microscope image of the pPINs and schematic diagram of the photonic PCR process in hydrogel matrix of pPIN. (B) Temperature curves with different duration time of denaturation and annealing/extension, and their corresponding normalized fluorescence curves of ultrafast pPIN-based qPCR. (C) Serial snapshots of the pPIN with (above) and without (below) the DNA template after ultrafast 5 min photonic qPCR. Reproduced with permission from [30]. Copyright 2022, The Authors, published by American Chemical Society.

Kim’s team [30] demonstrated a photonic real-time PCR platform in hydrogel microparticles. As shown in Fig. 10A, the photothermal hydrogel microparticles of primer-immobilized network (pPIN) were prepared by embedding reduced graphene oxide (rGO) and specific PCR primers into a polyethylene glycol (PEG) hydrogel. Each pPIN particle was a separate vessel that was ready for PCR. The only thing needed to do was introducing the mixture of target DNA and PCR Mastermix into the chamber of the pPIN. After that, the mineral oil was injected to surround and isolate the pPINs, and a 1-W 800-nm laser was used as the excitation light to drive the in-particle photonic PCR. Since the photothermal reaction is confined to nanoliter volume (~100 nl) of particles and the surrounding solution is always kept at 21 to 23 °C, it is easy to get a rapid heating (22.0 ± 3.0 °C/s) and cooling (23.5 ± 2.6 °C/s) rates during thermal cycling. As shown in Fig. 10B and C, by optimizing the duration time of denaturation, annealing, and extension, the overall PCR time can be reduced from 20 to 5 min without compromising amplification efficiency (i.e., maintaining a consistent real-time PCR curves). In this work, rGO improves its dispersion through the repulsion between its adsorbed negatively charged DNA, reducing the effect of rGO in the solution under light for easy rapid aggregation and random condensation, thereby achieving a uniform temperature distribution in a single pPIN. In addition, by reducing the reaction volume (~100 nl) and integrating real-time in situ fluorescence measurements, the pPIN-based qPCR is able to perform 40 thermal cycles in less than 5 min.

**Fig. 11.**
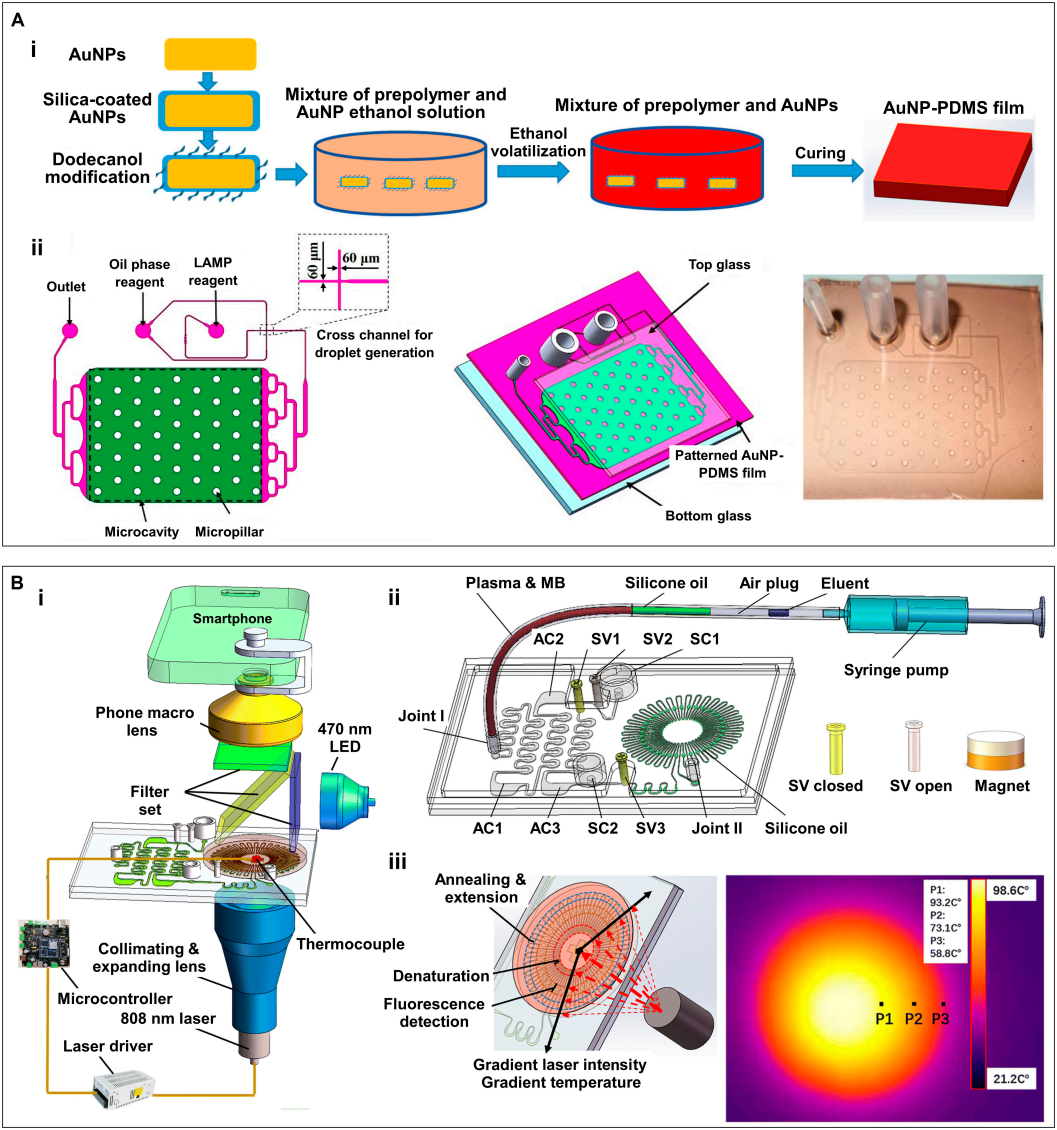
The AuNP-PDMS film and the smartphone-based microfluidic nucleic acid quantification system based on it. (A) (i) Schematic diagram of the manufacturing process of the AuNP-PDMS film. (ii) Schematic and photo of the microfluidic chip based on the patterned AuNP-PDMS film. Reproduced with permission from [189]. Copyright 2020, The Authors, published by MDPI. (B) (i) Diagrammatic illustration of the smartphone-based microfluidic nucleic acid quantification system. (ii) Schematic illustration of the microfluidic chip contains a sample preprocessing submodule based on sequential injection of immiscible reagents and a CF-PCR submodule with ring-arranged microchannels. (iii) Schematic diagram of the GPPT heater and the IR image of the CF-PCR submodule under laser irradiation. Reproduced with permission from [252]. Copyright 2022, The Royal Society of Chemistry.

In another work, Peng’s team [189] incorporated the silica-coated and dodecanol-modified AuNPs into PDMS substrate and cured the mixture to form AuNP-PDMS films (Fig. 11A, i). They improved the photothermal conversion efficiency of the AuNP-PDMS mixture by adjusting the concentration and morphology of AuNPs, resulting in uniform and stable heating under NIR LED (808 nm) irradiation. After that, soft lithography, chambers, and channels were fabricated on the prepared AuNP-PDMS film, which was then sandwiched between 2 glass substrates to create a sandwich microfluidic chip of “glass–PDMS–glass” structure (Fig. 11A, ii). This chip was capable of performing droplet-based sample partitioning and LED-driven photothermal LAMP nucleic acid amplification.

Building on the previous research, as illustrated in Fig. 11B (i), the team integrated the PPT effect with continuous-flow PCR (CF-PCR) to design and fabricate a microfluidic nucleic acid quantification system that integrates sample preprocessing and smartphone result reading. Figure 11B (ii) shows a schematic diagram of this microfluidic chip system. The chip primarily includes a sample preprocessing module based on sequential injection of immiscible reagents and a CF-PCR submodule with ring-arranged microchannels that are made of the AuNR-PDMS mixture. As shown in Fig. 11B (iii), utilizing the combination of PPT effect of AuNRs and the radial gradient intensity of the laser spot, a thermal gradient for gradient plasmonic photothermal (GPPT) CF-PCR is formed in a 32-mm-diameter coin-sized area. The AuNR-PDMS photothermal scheme used in this system effectively eliminates the inhibition of PCR by nanomaterials, while the thermal gradient and annular thermal microfluidic layout design avoid the dynamic heating of traditional PCR. With smartphone fluorescence reading, this system can complete nucleic acid sample purification and enrichment within 14 min and achieve hepatitis B virus (HBV)-DNA quantification from sample to answer in 37 min.

In conclusion, this chapter categorizes the recently reported photonic PCR technologies into 3 types based on their specific implementation strategies. We provide a detailed overview of the latest research achievements in each category above, focusing on the selection and preparation of photothermal materials, the fabrication of photothermal devices (consumables), and the development of photothermal cycling systems. It is believed that through this chapter, readers can get a more comprehensive and clear understanding of the applications of photothermal nanomaterials in photonic PCR.

Moreover, in this chapter we also summarize the current challenges of photonic PCR. Although the heating method based on the photothermal conversion effect significantly accelerates the thermal cycling process and shortens the reaction time, issues such as the inhibition of PCR amplification by nanomaterials and the quenching effect of nanomaterials on fluorescence, which affect the detection sensitivity and accuracy, still lack systematic solutions. As a result, photonic PCR remains some distance away from large-scale clinical application.

## Key Technologies in Photonic PCR

### Excitation light for photonic PCR

The essential distinction between photonic PCR and conventional PCR lies in their heating sources and methodologies. Currently, most mature commercial PCR instruments utilize TECs as heating/cooling elements, regulating the temperature of liquid in reaction tubes through indirect “metal-bath” heating, making it difficult to reduce the power consumption while also limiting the speed of temperature changes in the liquid. Photonic PCR technology abandons the traditional “metal-bath” heating model, enabling direct and rapid heating of PCR reagents through the synergy between excitation light sources and photothermal nanomaterials. Both previously mentioned planar-heating and volumetric-heating photonic PCR systems exhibit lower power consumption, smaller footprints, and higher heating efficiency compared to conventional PCR instruments.

The photonic PCR system is essentially a thermal cycling control device that uses photothermal nanomaterials as a carrier and is constructed by integrating a matching excitation light source. Therefore, the selection of excitation light sources, optical design, and illumination control directly determine the accuracy of PCR reagent temperature control and thermal cycling rates. Different types of photothermal nanomaterials possess distinct absorption spectrum characteristics. Referring to Tables 1 and 2 showcasing excitation light sources for various nanomaterials, we can summarize optimal excitation sources for common photothermal materials. AuNFs typically require blue light at ~450 nm and gold nanospheres (AuNSs) exhibit peak absorption at ~550 nm, while AuNRs generally utilize IR light >800 nm. At the same time, for the same type of material, its dimension and shape also affect light absorption. Son et al. [28] revealed through simulations that absorption spectra of AuNFs vary significantly with thickness. When illuminated by 450-nm peak-wavelength LEDs, photothermal conversion efficiency increases with thickness within the 10- to 120-nm range. Lee et al. [80] achieved significantly enhanced visible light absorption by constructing plasmonic nanoarrays, enabling ultrafast thermal cycling control using high-power white LEDs. Thus, selecting excitation sources precisely matched to the optical absorption properties of employed nanomaterials is imperative for optimal photothermal conversion.

In photonic PCR, excitation sources primarily fall into 2 categories: LED and laser. LEDs operate via spontaneous emission, utilizing the electroluminescence effect in forward-biased PN junctions of semiconductors. When electrons and holes recombine near PN junctions, electrons transition from higher to lower energy levels, releasing energy as photons. LED emission is incoherent with broad spectra [190]. On the other hand, lasers function through stimulated emission and high-energy electrons excited by incident photons transition to lower energy levels, emitting photons identical to the incident light. Consequently, laser emission is coherent with exceptional monochromaticity and narrow divergence angles [191]. Comparing their emission principles and characteristics reveals that laser sources offer higher power density and narrower spectral width, delivering superior heating efficiency. Laser scanning technology enables point-by-point excitation for high-throughput samples, effectively mitigating edge effects. However, laser diodes incur higher costs and require integrated heat dissipation modules, imposing compromises in device miniaturization and portability. In contrast, LEDs present advantages including low power consumption, low cost, and ease of integration. Their drawbacks—excessive spectral width, wide emission angles, lower power density, and poor spot uniformity leading to suboptimal heating efficiency—can be mitigated through nanomaterial optimization and ring-array configurations of multiple LEDs [156]. Upon finalizing photothermal nanomaterials and selecting matched excitation sources, designing driver circuits and control algorithms becomes essential for achieving rapid and precise photothermal control. The luminous efficiency of LEDs is positively correlated with current, yet their volt–ampere characteristic is nonlinear, and the working current exhibits an exponential relationship with the applied voltage. Additionally, as a semiconductor device, LEDs exhibit negative temperature characteristics where their forward voltage decreases with increasing temperature [192]. Therefore, multi-level closed-loop control integrating current, temperature, and light intensity feedback is required for precise LED control [193]. As for laser, the driver circuits demand high-speed response and interference resistance, employing high-speed optocouplers to isolate control signals from power stages while optimizing differential amplifier designs to suppress common-mode noise. Concurrently, thermal management using aluminum-based heat sinks with active cooling minimizes laser wavelength drift. Regarding control algorithms, beyond classical proportional integral derivative control, neural network-based algorithms can predict light intensity and dynamically adjust excitation power to achieve accurate temperature control.

The core innovation of photonic PCR lies in replacing the indirect metal-bath heating of conventional PCR with a photothermal nanomaterial–excitation light coupled system, fundamentally overcoming technical bottlenecks such as low efficiency and thermal conduction delays. Excitation light selection must strictly align with the absorption spectra of photothermal materials, while nanomaterial morphology engineering maximizes photothermal conversion efficiency. Regarding technical pathways, laser sources, with high power density and narrow spectra, better suit high-throughput precision temperature control scenarios. However, its high cost and cooling requirements limit its portability, whereas LED sources, through ring-array configurations and spectral optimization of the materials, enable homogeneous heating with low power consumption and cost, making them ideal for miniaturized devices. Regardless of light source type, driver circuits and control algorithms must satisfy stringent requirements of millisecond response and ±0.1 °C stability—critical for realizing photonic PCR’s ultrafast and low-power advantages. Future iterations demand further co-optimization of “material–light source–temperature control” triad designs to advance photonic PCR clinical translation under World Health Organization’s (WHO) ASSURED (affordable, sensitive, specific, user-friendly, rapid and robust, equipment-free, delivered) criteria.

### Temperature sensors for photonic PCR

Regardless of the form of heating, collecting sample temperature as a feedback signal is necessary for thermal control. Therefore, accurate temperature monitoring is the key to thermal cycling control of PCR [194–196]. Common temperature sensors include thermocouples and thermistors. In traditional PCR devices, such sensors are usually placed closely against the container wall or a metal block to get the approximate temperature of the heated liquid [74,197,198]. However, in photonic PCR, the heating method shifts from contact thermal conduction to noncontact direct heating of the liquid using light without the need for heat-conducting metal blocks. Additionally, to enhance the rate of temperature change, in most research, reaction vessels are often designed to be thin and narrow, making it difficult to install traditional temperature sensors [199]. Besides, inserting temperature sensors directly into the liquid for temperature measurement poses a risk of contaminating the sample. Therefore, temperature collection presents one of the challenges in photonic PCR [200–202].

As is well known, all objects emit electromagnetic radiation in the IR spectrum with an intensity proportional to their surface temperature. Utilizing this principle, IR thermometers receive and measure the IR radiation emitted by objects, and then the surface temperature of the object can be calculated based on the radiation intensity [203–205]. Due to its noncontact nature, rapid response, wide measurement range, and high accuracy, IR thermometry is widely used in industries, medical science, and scientific research. Considering the noncontact heating, fast heating and cooling rates, and small volume of heated liquids in photonic PCR, IR thermometry is prioritized and extensively applied for temperature measurement in photonic PCR processes [126,156,206].

**Fig. 12.**
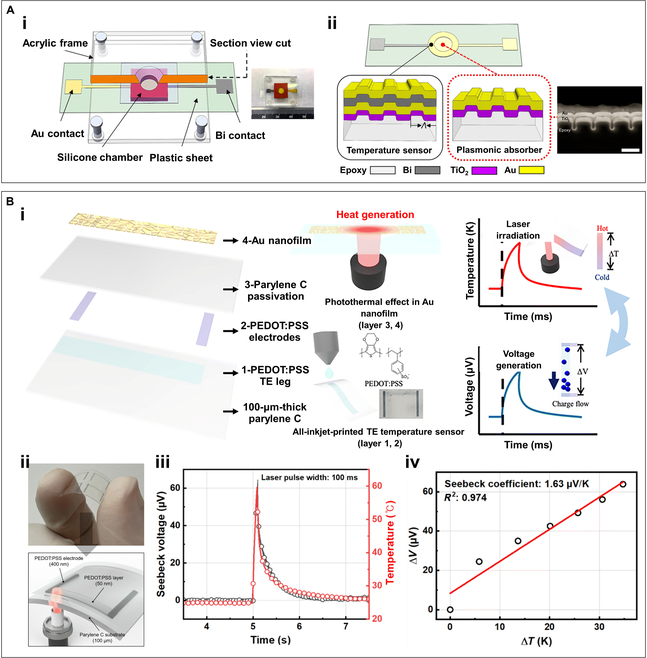
Novel temperature sensors for ultrafast photonic PCR. (A) (i) Schematic diagram and photograph of the phoththermal PCR device with an integrated on-chip temperature sensor. (ii) Schematic illustration and SEM image of the cross-section cut of the plasmonic chip, in which the central dot consists of a Au/TiO_2_ grating for photonic heating, while the outer ring includes an extra stack of Au/Bi film as the thermocouple for temperature measurement. Reproduced with permission from [210]. Copyright 2020, Wiley-VCH. (B) (i) Conceptual diagram of the all-inkjet-printed TE transparent temperature sensor with high temporal resolution and its working principle. (ii) Photograph and schematic illustration of the flexible PEDOT:PSS-only TE temperature sensor on a flexible parylene C substrate. (iii) Transient characteristic curves of the temperature value and Seebeck voltage changes of the sensor upon 100-ms-long photothermal effect generation. (iv) Liner fitting plot of temperature change versus Seebeck voltage change value. Reproduced with permission from [211]. Copyright 2023, The Royal Society of Chemistry.

However, IR thermometry also has limitations. For instance, it can only measure the surface temperature of objects, which makes it inconvenient for measuring temperatures inside objects or when obstacles are present. Additionally, the accuracy of IR thermometry is closely related to the measurement distance and angle [207–209]. Thus, sensors must be carefully positioned and calibrated to obtain accurate temperature readings. Therefore, in addition to IR thermometry, researchers are also trying to develop other temperature measurement methods to accurately monitor the temperature of reagents during photonic PCR. Lu and colleagues [210] developed a plasmonic thermal cycler that integrates photonic heating elements and temperature sensors into the same chip to accurately monitor reagent temperature. As shown in Fig. 12A, the heating region of the dual-function sensor chip consists of a one-dimensional PET plastic grating and multiple thin-film coatings. The multilayer coatings, from bottom to top, include titanium dioxide (TiO_2_), a 90-nm-thick gold (Au) film, a 150-nm-thick bismuth (Bi) coating, and an extra 50-nm-thick Au layer on top. The TiO_2_ and Au films together form the plasmonic resonance layer, matching the wavelength of an 808-nm laser. On the other hand, the Au film also acts as the positive electrode metal side of a thermocouple, forming an Au–Bi junction with the Bi coating, from which the solution temperature is obtained by measuring the output voltage across the junction. In addition, the top Au layer is used to protect the Bi layer from corrosion and destruction by the PCR reagents. With the on-chip Au–Bi thermocouple constructed, this plasmonic thermal cycler can complete a PCR thermal cycle in approximately 2.5 min and has successfully achieved multiplex real-time PCR amplification of antibiotic resistance genes. This work integrates multiple thin-film coatings on the same chip to simultaneously construct a photothermal heater and temperature sensor, achieving effective monitoring of reagent temperature while heating PCR reagents, providing a new approach for the integration of nanofilm-based planar-heating photonic PCR devices.

Kang’s team [211] introduced a novel concept of directly measuring optothermal effects using transparent thermoelectric (TE) temperature sensors for the first time. They utilized the Seebeck effect to convert temperature gradients into electrical potential. Based on this principle, they successfully measured photothermal effects occurring within submicrometer distances (500 nm) with ultrafast response speed and super high temporal resolution (100 μs). Poly(3,4-ethylenedioxythiophene) polystyrene sulfonate (PEDOT:PSS), known for its high Seebeck coefficient, biocompatibility, and mechanical flexibility, was chosen as the material to construct the TE temperature sensor. Through optimization, high optical transmittance (>95%) was achieved in the range of 380 to 850 nm, minimizing attenuation of the incident light. Figure 12B (i) illustrates the design concept of the transparent temperature sensor utilizing the TE effect, as well as its implementation for measuring optothermal effects. More specifically, low-temperature inkjet printing was used to deposit PEDOT:PSS TE legs (layer 1) and electrodes (layer 2) onto a 100-μm-thick parylene C substrate to construct the transparent temperature sensor, which measured the temperature of photothermal effect induced by the gold nanofilms (layer 4). Figure 12B (ii) shows photograph and schematic of the PEDOT:PSS TE temperature sensor constructed on a parylene C flexible substrate. Figure 12B (iii) shows the Seebeck voltage curve of the sensor under 100-ms-long photothermal effect, indicating that the sensor has a very fast response time. At the same time, there is a good linear relationship between the temperature value and the Seebeck voltage, which is very close to the temperature data of the thermal imaging IR camera (Fig. 12B, iv). There is reason to believe that the high temporal resolution transparent temperature sensor based on the TE effect developed in this work will open up new opportunities for temperature measurement in ultrafast photonic PCR.

Accurate and noninvasive temperature monitoring presents a significant challenge in photonic PCR systems, primarily due to the shift from contact heating to noncontact photonic heating, the miniaturization of reaction vessels, and the imperative to avoid sample contamination. While IR thermometry, leveraging its noncontact nature and rapid response, has become the predominant method for surface temperature measurement in photonic PCR, its inherent limitations—such as measuring only surface temperature and susceptibility to measurement distance and angle—necessitate careful calibration and positioning. Future advancements in photonic PCR temperature sensing will likely focus on further enhancing the sensitivity, spatial resolution, and multiplexing capabilities of such integrated sensors, potentially incorporating novel materials and transduction mechanisms, to enable even more precise thermal control and facilitate complex applications like ultrafast real-time qPCR within highly integrated microfluidic systems.

### Amplification product detection for photonic PCR

PCR-based nucleic acid detection includes 2 parts: exponential amplification of target sequences and detection of amplification products [212–215]. In the early days of PCR technology, agarose gel electrophoresis was used to separate the amplified nucleic acid fragments, and the concentration of the products was qualitatively judged based on the brightness of the electrophoresis bands. This method requires the extraction of products and additional electrophoresis steps after the amplification, increasing the overall detection time and making quantitative analysis impossible. Consequently, researchers incorporated fluorescent groups (including fluorescent probes or dyes) into conventional PCR system to achieve real-time in situ monitoring of the amplification products by measuring the fluorescence intensity of the fluorescent groups, which is known as real-time qPCR. Furthermore, quantitative analysis of the initial template can be achieved through standard curves. Real-time qPCR technology not only has greater advantages in sensitivity and specificity but also effectively reduces the risk of cross-contamination by completing the detection operation without the need for opening the lid. Therefore, real-time qPCR remains the primary method for nucleic acid quantification analysis [216–218].

**Fig. 13.**
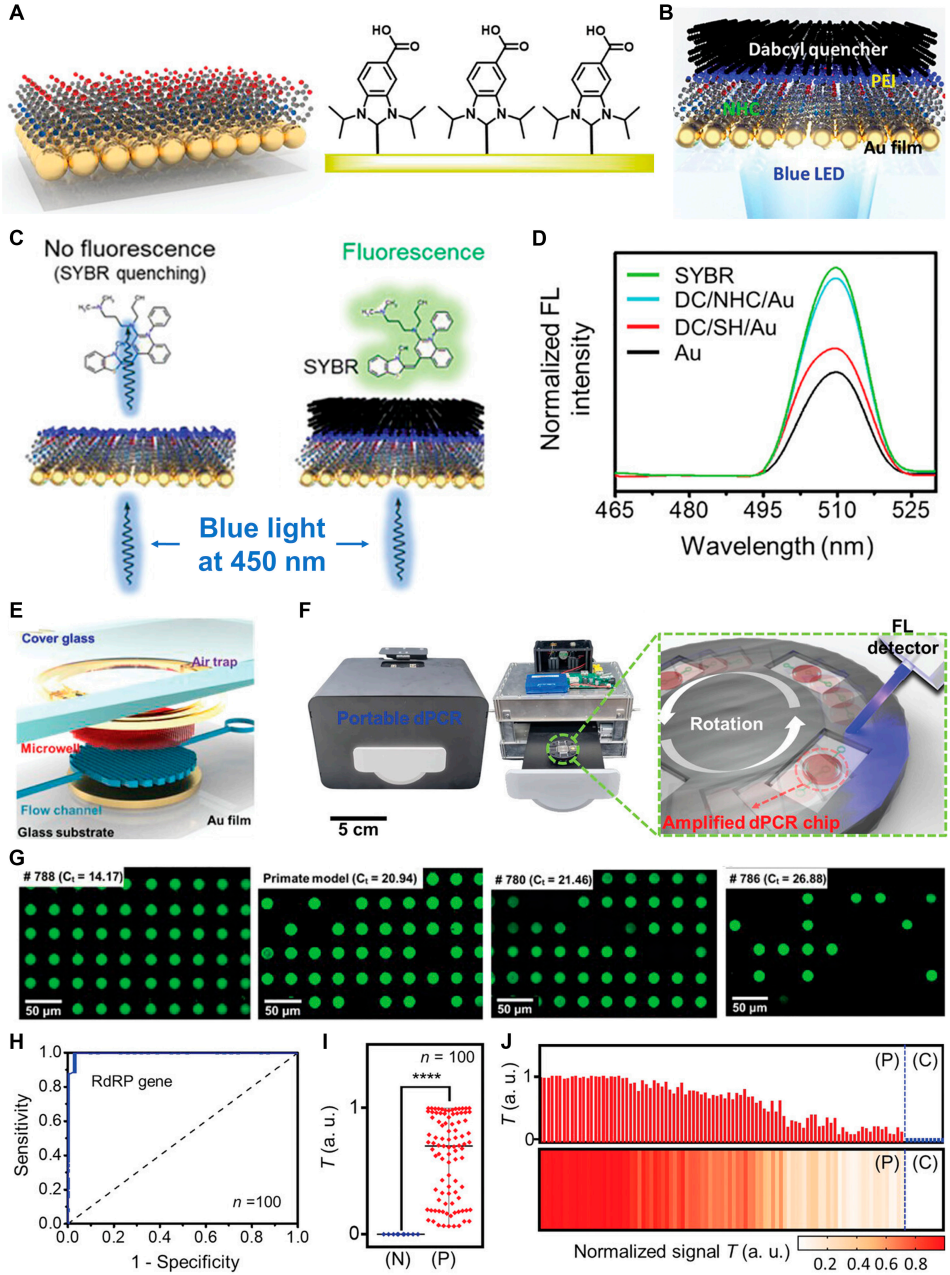
The ultrafast portable photonic dPCR system based on NHC SAM. (A) 3D structures of NHC-introduced Au and the chemical structural of NHC. (B) Schematic illustration of the DC/NHC-introduced Au film exposed on the blue LED. (C) Schematic illustration of the fluorescence quenching (left) and anti-quenching (right) effect of the NHC-introduced Au film without/with DC. (D) Fluorescence intensity of SYBR on different Au films after PCR cycle. (E) Schematic diagram of the 3D structures of the multilayer dPCR chip, which consists of an Au film and microfluidic flow channel sandwiched between a glass substrate and a cover glass. (F) Photography of the portable photonic dPCR device equipped with a high-velocity fluorescence scanner (in green dotted box). (G) Fluorescence images of dPCR results of various clinical sample with different Ct value. (H) ROC curve of the detection result of SARS-CoV-2 from 100 patients by the photonic dPCR. (I) *t* Test analysis based on the ROC curve (^****^P < 0.0001). (J) Waterfall plots for the detection result of clinical samples of SARS-CoV-2. Reproduced with permission from [88]. Copyright 2023, Wiley-VCH.

As mentioned previously, the wavelength of the heating excitation light for the planar-heating photonic PCR based on AuNFs overlaps with the wavelength of the excitation light for commonly used fluorescent dyes. Studies have shown that the transmitted residual excitation light can cause fluorescence quenching, thereby affecting the accuracy and repeatability of quantitative analysis results [219,220]. In some studies, the introduction of compounds containing thiol groups has been used to improve the fluorescence quenching phenomenon. However, the thiol groups dissociate from Au upon heating, limiting their application in PCR. To address this challenge, as shown in Fig. 13A, Kwon’s team [88] synthesized an interface chemical substance with a novel structure, N-heterocyclic carbene (NHC), which can form covalent bonds with the (111) plane of gold under thermal stress, resulting in the formation of self-assembled monolayers (SAMs) on the Au film. Experiments have shown that the NHC-introduced Au film possesses high-speed thermal cycling capabilities (with heating and cooling rates of 8.75 and 17.5 °C/s, respectively) and excellent thermal stability, making it an ideal material for planar-heating photonic PCR. To prevent the quenching of the fluorescence dye caused by transmitted residual excitation light, they introduced Dabcyl quencher (DC) to further block the transmitted residual light through the NHC/Au film (Fig. 13B and C). Figure 13D shows the fluorescence intensity of SYBR Green on different Au films, in which the DC/NHC-introduced Au film significantly reduced the quenching of SYBR Green, with its fluorescence intensity increased by over 90% compared to the original Au film. Using the constructed DC/NHC-introduced Au films with a PDMS-based multilayer fluid channel and cover glass, they developed a dPCR chip (Fig. 13E) and designed a portable device integrating a high-speed rotating photonic scanner and photonic dPCR system (Fig. 13F). This device can perform rapid thermal cycling amplification and fluorescence scanning detection of 40 cycles within 15 min. Figure 13G presents the fluorescence images of photonic dPCR results for clinical samples with different concentrations. The detection performance of the photonic dPCR system was validated by comparison with RT-PCR test results for clinical SARS-CoV-2 samples. Figure 13H presents the receiver operating characteristic (ROC) curve of the photonic dPCR results for 100 samples, revealing a system sensitivity of 99%, specificity of 96.8%, and accuracy of 95.3%. The accuracy of this portable system was further confirmed by the area under the ROC curve (AUC) parameter (0.996), demonstrating high concordance with RT-PCR in clinical diagnostic performance (Fig. 13I). The waterfall plot illustrates the distribution of all clinical sample results (*n* = 100) based on *t* test analysis (Fig. 13J). These findings indicate that the portable photonic dPCR system exhibits exceptional diagnostic performance, fully complying with the POCT criteria for suspected COVID-19 cases of the WHO.

Fluorescence detection, regarded as the “gold standard” method for evaluating PCR results, offers high sensitivity and specificity [221–223]. However, fluorescence detection requires complex and expensive fluorescence excitation and scanning detection equipment, which poses challenges for low-cost testing needs. Compared with fluorescence detection method, colorimetric sensing is an emerging detection method that uses color changes for analysis [224–226]. This method can achieve qualitative or quantitative analysis by direct visual observation or using a spectrophotometer [227], having advantages such as ease of operation, visual readability, and no need for complex precision instruments, making it widely applied in the development of various low-cost POC biosensing platforms [228–230]. In recent years, the pandemics of infectious diseases like COVID-19 have highlighted the urgent demand for low-cost detection methods, leading to increased interest in combining nucleic acid amplification technology with colorimetric sensing [231–233].

**Fig. 14.**
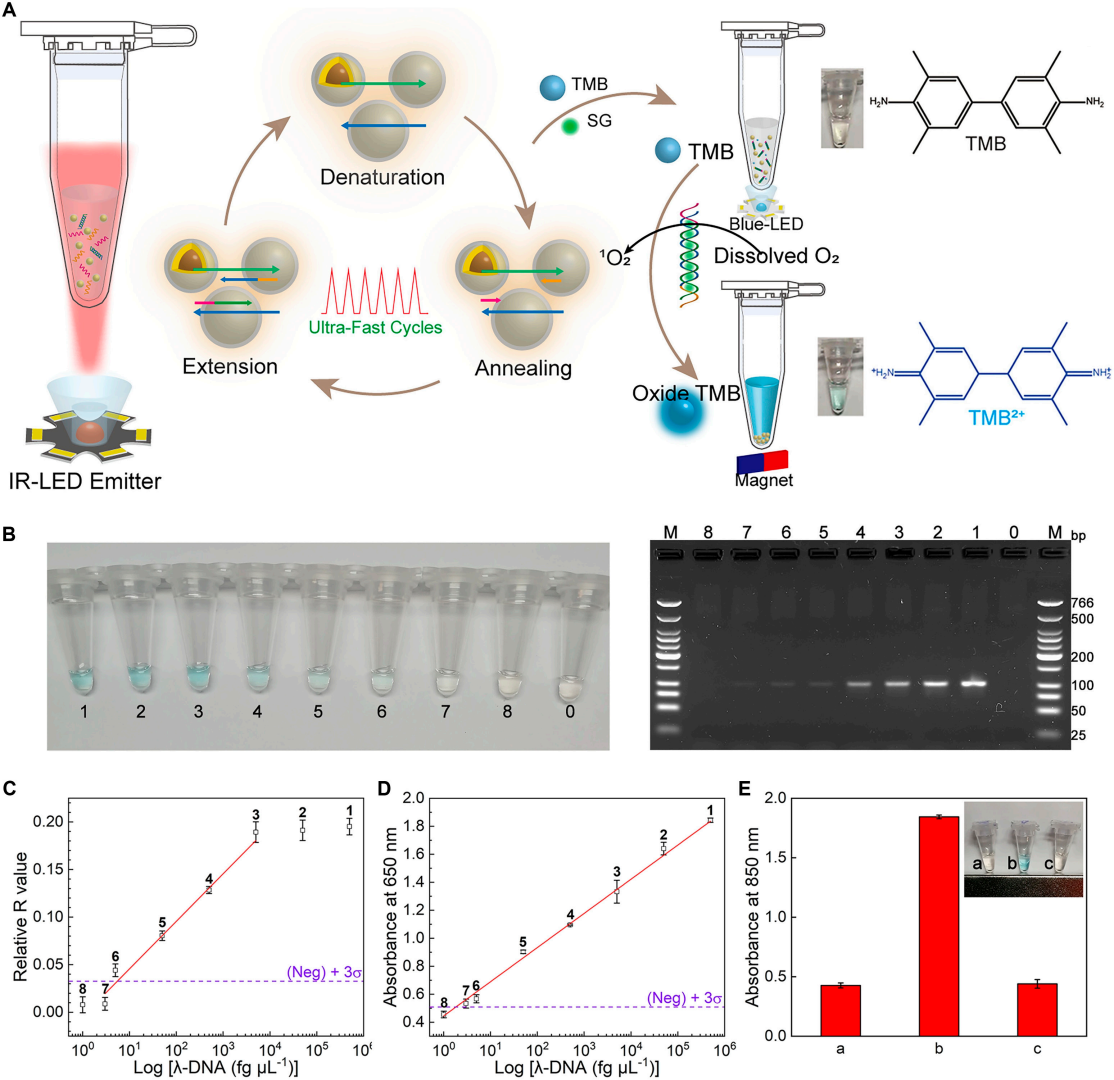
The PPT-cPCR technique based on TMB oxidation method. (A) Schemes of PPT-cPCR process. (B) Photographs (left) and agarose gel electrophoresis images of PPT-PCR amplification results of serially diluted target DNA. (C) Plots of relative *R* value of the photograph of the samples after amplification against λ-DNA target concentration. (D) Plots of the absorbance of the sample solution against λ-DNA target concentration. (E) Photograph and absorbance at 850 nm of 3 different samples after amplification. a, negative control; b, 0.5 ng μl^−1^ λ-DNA target; c, 0.2 ng μl^−1^ other DNA sequence. Reproduced with permission from [234]. Copyright 2021, Elsevier.

Up to now, a wide range of colorimetric sensing assays has been designed for biomarker detection, among which 3,3′,5,5′-tetramethylbenzidine (TMB) has been widely used due to its color change upon oxidation by peroxidases. Additionally, the oxidation of TMB can also be catalyzed by reactive oxygen species (ROS). For example, a color change of TMB will produce in the presence of singlet oxygen, which is produced from the dissolved oxygen through photocatalytic activity of double-stranded DNA-SYBR Green I (DNA-SG1) complex under the illumination of blue light. Inspired by this, Jiang et al. [234,235] proposed a novel plasmonic photothermal colorimetric PCR (PPT-cPCR) technique based on TMB oxidation method and applied it to the detection of dengue virus. Figure 14A shows the amplification process of PPT-cPCR based on PMNs as well as the oxidation process of TMB by photocatalytic activity of the amplified DNA-SG1 complex. In this system, the PMNs act as nano-photothermal devices to achieve thermal cycling under IR LED irradiation, completing the amplification of target DNA. Subsequently, SG1 binds to the target DNA to form the DNA-SG1 complex, which produces singlet oxygen from dissolved oxygen under blue light irradiation. After that, TMB is oxidized, resulting in a color change. Finally, qualitative results can be determined by visual observation, or quantitative information can be obtained through a smartphone and spectrophotometer. Figure 14B presents photographs of PPT-PCR amplification results of serially diluted target DNA and their agarose gel electrophoresis images. By using PMNs (12.3 OD), the target amplification process, which contains 30 thermal cycles, can be completed within 5 min. Subsequently, TMB solution is added into the solutions followed by the irradiation with blue LED. Then, qualitative determination of target concentration can be observed based on the color change of the mixture, specifically from apricot color in the absence of target DNA to blue in the presence of target DNA, with increasing blue intensity corresponding to higher concentrations. Quantitative detection results can be obtained through image capture by a smartphone (Fig. 14C) and absorbance measurement by an ultraviolet–visible (UV–Vis) spectrophotometer (Fig. 14E). Figure 14E evaluates the selectivity of the PPT-cPCR using no-template control samples and nonspecific targets, demonstrating that significant color change occurred only in samples containing the target DNA, demonstrating the excellent selectivity of the PPT-cPCR system.

**Fig. 15.**
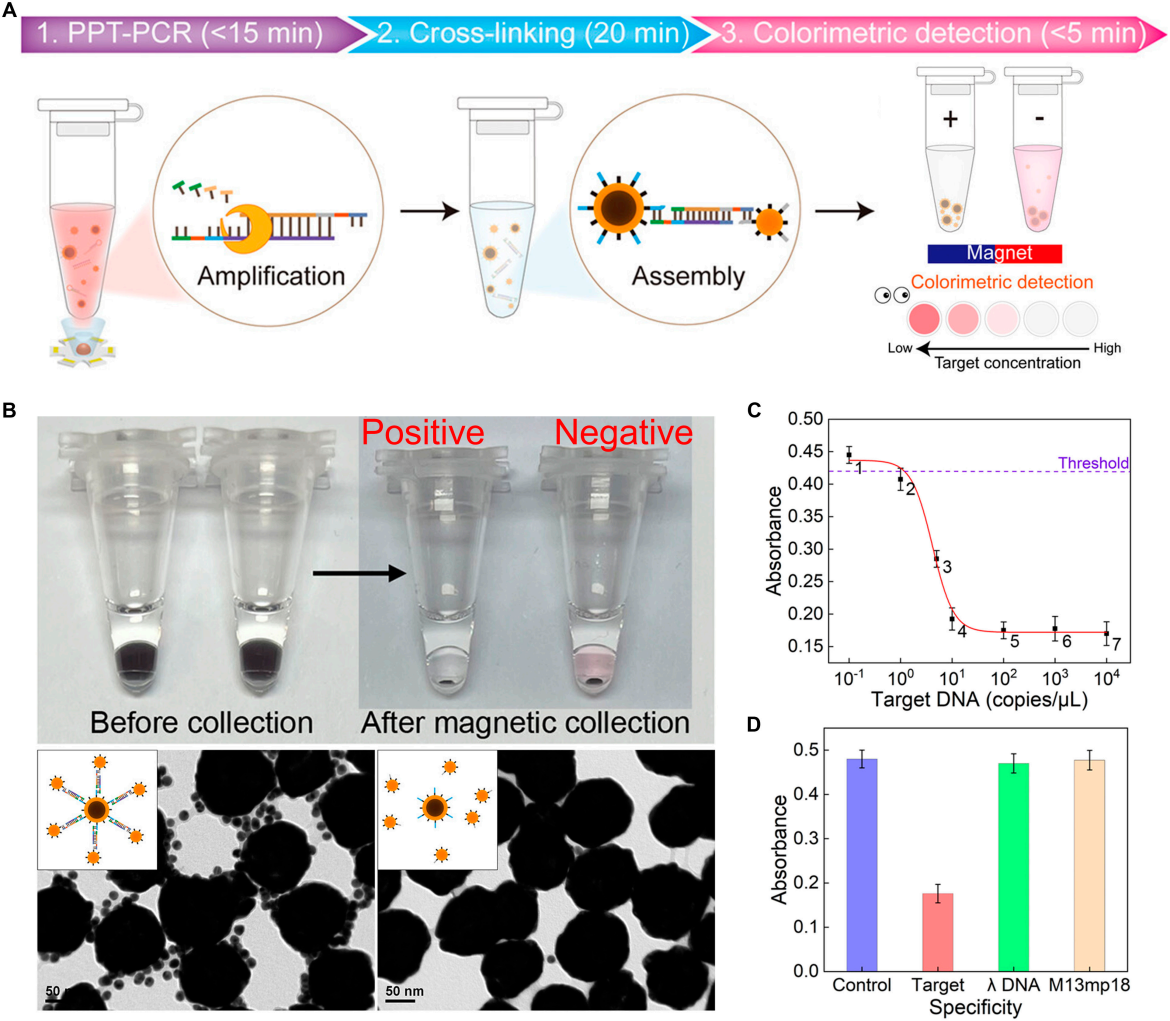
The PPT-ccPCR method for rapid and convenient nucleic acid detection. (A) Schematic illustration and workflow of the PPT-ccPCR. (B) Photograph of the positive and negative samples before and after magnetic collection, and the TEM images of the positive sample with sandwich assembly structure and the negative sample without the assembled structure. (C) Absorbance curve of the supernatant of PPT-ccPCR products with different target concentrations. (D) Assessment results of selectivity of the PPT-ccPCR. Reprinted with permission from [236]. Copyright 2023, American Chemical Society.

As shown in Fig. 15A, Lee’s team [236] proposed another novel plasmonic photothermal cross-linking colorimetric PCR (PPT-ccPCR) for rapid and convenient nucleic acid detection. In this work, DNA-modified PMNs and DNA-modified AuNP cross-linking assembly was applied, in which PMNs are used for PPT heating and assembly collection, while AuNPs are used for recognition and color indication. To be specific, PMNs first serve as nano-photothermal devices to carry out photon-driven ultrafast thermal cycling amplification of the target sequence under IR light irradiation. Subsequently, the DNA-modified PMNs and AuNPs hybridize with the PCR amplicon, forming a PMN–DNA–AuNP sandwich assembly. Due to the presence of PMNs, the assembly can be collected by an external magnetic field, resulting in a colorless supernatant. On the contrary, if the target nucleic acid fragments are absent in the reaction system, the sandwich assembly does not form, so the magnetic field only collects the PMNs, while leaving the AuNPs dispersed in the solution, giving it a pink color. Figure 15B shows photographs of PPT-ccPCR results for positive and negative samples before and after magnetic separation, along with TEM images of the assembled (left) and non-assembled (right) structures. By optimizing the concentration of the coupling probes, this sensor can achieve visual detection as low as 5 copies/μl within 40 min (Fig. 15C), and it demonstrates good specificity, effectively avoiding nonspecific amplification (Fig. 15D).

The amplification product detection technologies for photonic PCR are undergoing a transformation from single reliance on fluorescence to multi-adaptive scenarios. Fluorescence detection remains the preferred choice for clinical diagnostics due to its high sensitivity and specificity, maintaining its gold standard status through material interface modifications, system integration, and the application of image signal enhancement algorithms. In contrast, colorimetric methods, though less sensitive than fluorescence and prone to interference from pH and impurities, have expanded the boundaries of on-site testing via a more accessible approach. Future breakthroughs in photonic PCR product detection will require interdisciplinary collaboration in materials science, optics, and other fields, leveraging smart devices and artificial intelligence (AI)-based signal processing algorithms to advance molecular diagnostics toward faster, more accurate, and more accessible solutions.

## Integrated POC Photonic PCR System

The high sensitivity and specificity of PCR technology demonstrate its enormous potential for rapid pathogen identification, making it become the gold standard for infectious disease diagnosis. However, traditional benchtop PCR systems, using thermistors or Peltier elements for thermal cycling control, suffer from drawbacks such as heavy weight, large size, and high energy consumption, rendering them unsuitable for deployment in POC environments. Furthermore, slow ramp rates lead to excessively long detection times (~1 to 2 h), further limiting the application scenarios of PCR technology. The development of photonic PCR technology provides the technical foundation for the development of fast, portable, compact, and low-power PCR devices. Additionally, by integrating upstream sample purification and enrichment operations using microfluidic technology, an easy-to-use integrated nucleic acid rapid detection platform with a “sample in, result out” feature can be developed [237–240]. In this chapter, we will provide a detailed introduction to 3 different types of typical integrated nucleic acid detection platforms based on photonic PCR. Then, we will summarize the application prospects of photonic PCR in integrated nucleic acid platforms. At the same time, the commercialization of photonic PCR and the opportunities and challenges faced by clinical transformation will be discussed.

### One-pot photonic PCR system based on magnetic plasmonic nanoparticles

**Fig. 16.**
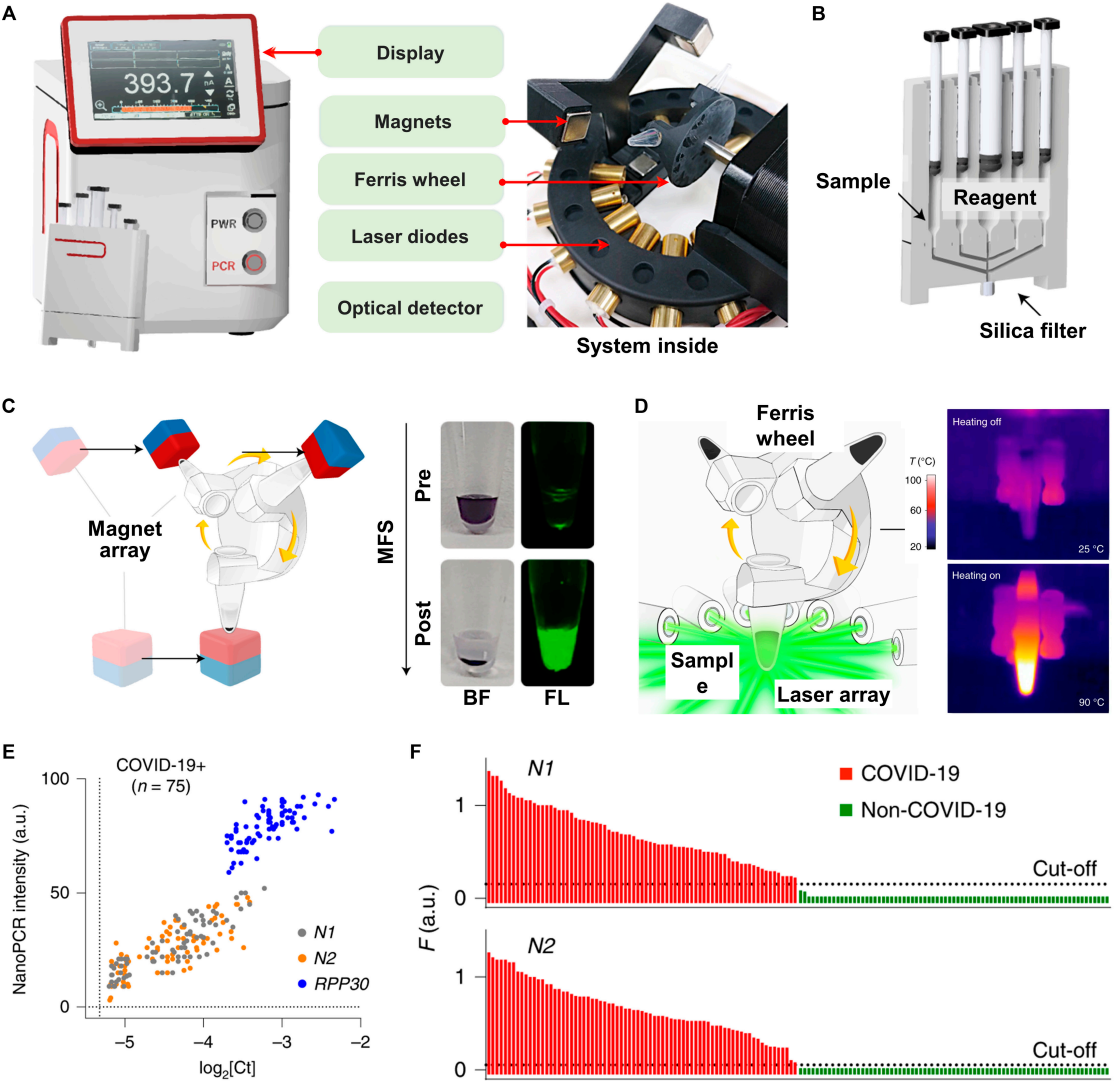
The rapid one-pot PCR system based on bifunctional magnetic plasmonic nanoparticles and RT-PCR technology. (A) Photograph of the compact nanoPCR instrument and its inside structure. (B) Photograph of the disposable RNA extraction kit. (C) Schematic illustration of the working principle of the movable magnet array for in situ detection of amplicons (left), as well as the brightfield and fluorescence photographs of an assay mixture before and after 3 min of magnetic separation (right). (D) Schematic diagram of the principle of thermal cycling control for multiple samples based on the low-powered laser diode circular array combined with the synchronized ferris wheel, as well as IR images of the PCR tube with the heater turned off (above) and on (below). (E) Assessment of detection consistency between nanoPCR and RT-qPCR for 3 genes in 75 positive COVID-19 samples. The results revealed a strong positive correlation between the raw fluorescence intensity of nanoPCR and the negative logarithmic values (−log_2_[Ct]) of RT-PCR Ct values. (F) Waterfall distribution of fluorescence intensity of N1 and N2 genes detected by nanoPCR in all of the 150 samples tested. Reproduced with permission from [128]. Copyright 2020, The Authors, published by Springer Nature.

Cheon’s team [128] reported a rapid one-pot PCR system based on bifunctional magnetic plasmonic nanoparticles and RT-PCR technology for the rapid detection of SARS-CoV-2, termed nanoPCR. As shown in Fig. 16A, the nanoPCR system includes an integrated device and a disposable RNA preparation kit, merging plasmonic thermal cycling with in situ fluorescence signal detection within a unified platform. In more detail, the integrated device primarily consists of a laser diode array arranged in a radial formation, a Ferris wheel-style rotating sample rack, a movable magnet, and a fluorescence excitation detection device. The use of low-power (80 mW) laser diodes arranged according to a prototype array achieves more uniform illumination compared to a single high-power laser. The Ferris wheel-style rotating sample rack can install multiple tubes simultaneously, and synchronization of sample rack rotation with the illumination schedule enables simultaneous PCRs for multiple samples (Fig. 16D). Rapid separation of PMNs is achieved through an external magnetic field formed by movable magnets, followed by in situ fluorescence signal detection using the fluorescence excitation detection device (Fig. 16C). Figure 16B displays the disposable kit used for RNA preparation, which contains multiple pre-loaded RNA extraction reagents in chambers and a silica gel filter for RNA capture. Based on the principle of solid-phase extraction, initiating plungers sequentially can carry out virus lysis, RNA adsorption, purification, and elution within 3 min. The entire apparatus is coordinated by a microcontroller unit, allowing automatic execution of reverse transcription, rapid PCR amplification, and in situ fluorescence detection at the push of a button. Figure 16E shows the nanoPCR’s diagnostic accuracy of 3 target genes from COVID-19 compared with traditional RT-qPCR. For the 75 clinical positive COVID-19 samples, a comparison was made between the raw fluorescence intensity measured by nanoPCR and the negative logarithm of cycle threshold (Ct) values (−log_2_[Ct]) obtained from RT-qPCR. The results demonstrated a strong positive correlation between the 2 methods for each target gene, with Pearson coefficient (*r*) values of 0.87 (*N1*), 0.78 (*N2*), and 0.70 (*RPP30*). Figure 16F displays the waterfall distribution of fluorescence intensity of N1 and N2 genes detected by nanoPCR in all of the 150 samples, and the diagnostic accuracy exceeded 99%.

### Nanophotonic light-driven chip integrated with cell lysis and PCR

**Fig. 17.**
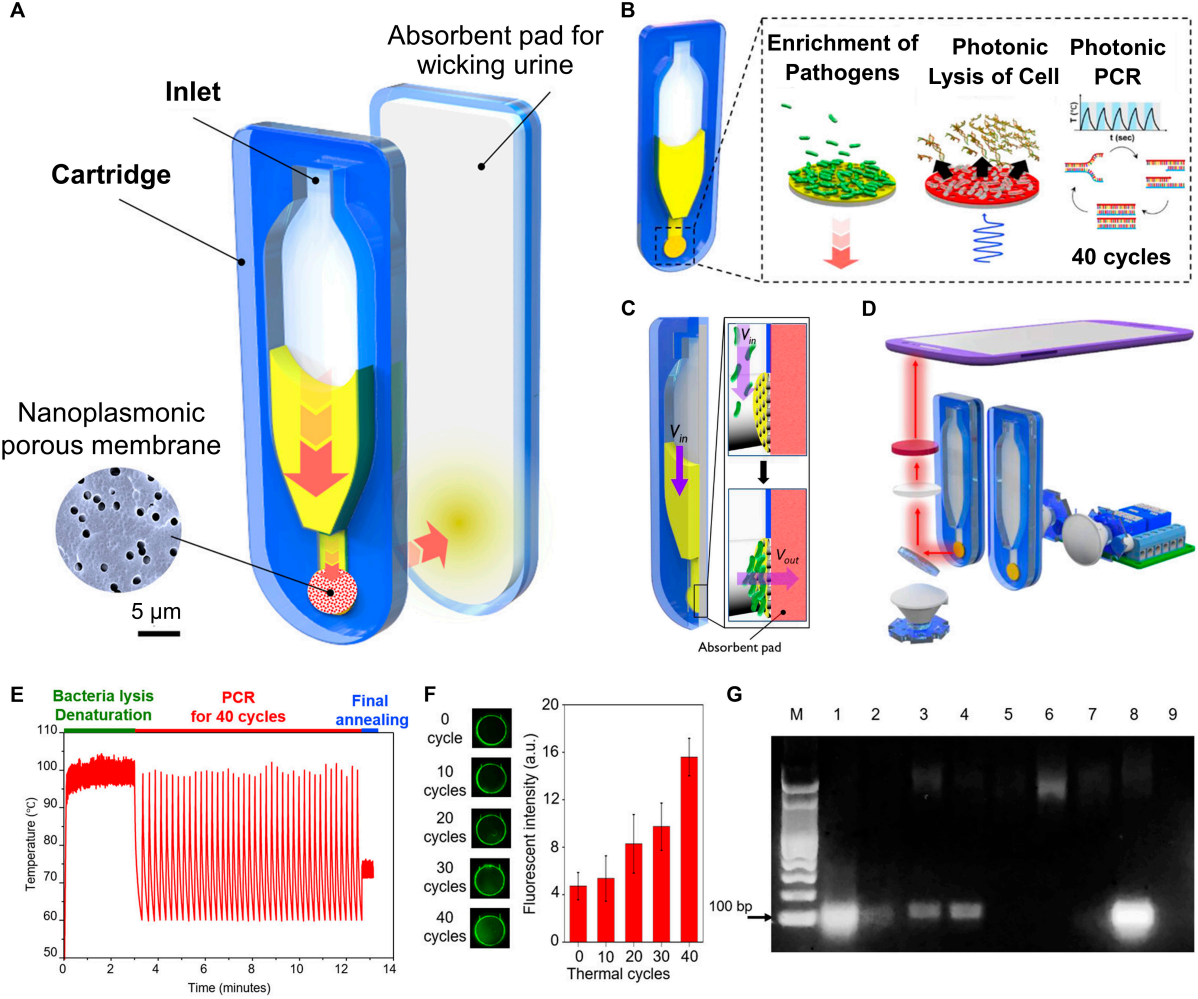
The nanophotonic light-driven integrated cell lysis and PCR on a chip with LIGHT for rapid and precise pathogen detection. (A) Schematic diagram of the structure and working principle of the LIGHT chip. (B) Three functions of the multifunctional Au-coated nanoporous membrane of the detection chamber. (C) Schematic diagram of the gravity-driven process for cell enrichment on the nanoporous membrane. (D) Conceptual vision of the LIGHT system with integrated fluorescence detection of smartphones. (E) Complete temperature profile encompassing bacterial lysis and PCR procedures. The entire procedure can be completed within 14 min, including 4 min for bacterial lysis and DNA denaturation followed by 40 thermal cycles for PCR amplification. (F) Fluorescence images (left) and fluorescence intensity (right) of SYBR Green dye during different stages of PCR amplification cycles. (G) Electrophoretic verification of PCR products from varying sample concentrations of the platform. Lanes 1 to 7, 10^6^ to 10 CFU/ml concentrations, respectively; lane 8, amplification of 10^6^ CFU/ml sample using conventional benchtop equipment; lane 9, negative control. Reprinted with permission from [86]. Copyright 2019, American Chemical Society.

Although the advent of PPT-PCR technology has significantly increased the speed of PCR amplification, nucleic acid detection involves cumbersome processes such as sample enrichment, nucleic acid purification, and amplification detection. Currently, most devices still require bulky heating modules, expensive lasers, and complex liquid control systems with pumps and valves, which undoubtedly increase the system’s complexity and cost, making them less ideal for POCT. Lee’s group [86] described a nanophotonic Light-driven Integrated cell lysis and PCR on a chip with Gravity-driven cell enrichment Health Technology (LIGHT) for rapid and precise pathogen detection. As shown in Fig. 17A, the main structure of the chip is made of PMMA and PC, primarily consisting of a cartridge main body in the front and a absorbent pad at the back, which are bonded together using double-sided adhesive tape. The cartridge main body mainly includes a big reservoir chamber and a dead-end PCR chamber that contains an Au-coated PC nanopore membrane with nanoplasmonic hole arrays. The device relies on a combination of gravity and the capillary action of the absorbent pad to autonomously drive liquid flow without the need for any external driving mechanism. The nanoplasmonic antenna is constructed by depositing a 5-nm titanium layer and an 80-nm Au layer on the PC porous membrane using electron beam evaporation. As shown in Fig. 17B, the nanoporous membrane can enrich pathogens through physical capture, achieving a 40,000-fold concentration of bacteria from a 1-ml sample within 2 min. It also serves as a photothermal conversion layer for photothermal cell lysis and photonic PCR heating. Figure 17C illustrates the working principle of the LIGHT system. After the sample is introduced into the chip, it flows into the detection chamber through the nanoporous membrane under the combined action of gravity and the capillary force of the absorbent pad. The bacteria in the sample are captured by the nanoporous membrane due to their size being larger than the pore size of the membrane, thereby achieving bacterial enrichment. Subsequently, the sample is heated using an LED light source, and the gold layer’s photothermal effect enables rapid bacterial lysis within 1 min. Finally, the photothermal effect of the nanoplasmonic antenna is used for rapid thermal cycling, completing 40 PCR cycles within 10 min to achieve DNA amplification. Figure 17E shows a complete temperature profile encompassing 4 min of bacterial lysis and DNA denaturation, and 10 min of 40 PCR cycles. SYBR Green dye was added into the reaction system, and the fluorescence intensity of the amplification product is observed under a microscope to detect the pathogen. Figure 17F shows the fluorescence images and fluorescence intensity of the detection chambers during different stages of PCR amplification cycles. As shown in Fig. 17G, this system demonstrated rapid detection of *E. coli* in clinical urine samples with a limit of detection (LOD) of 10^3^ colony-forming units (CFU)/ml. The team proposed, as shown in Fig. 17D, an envisioned system construction that integrated smartphone-based online fluorescence detection, truly achieving a fully automated “sample-in, result-out” process.

### Ultrafast plasmonic real-time RT-PCR system

**Fig. 18.**
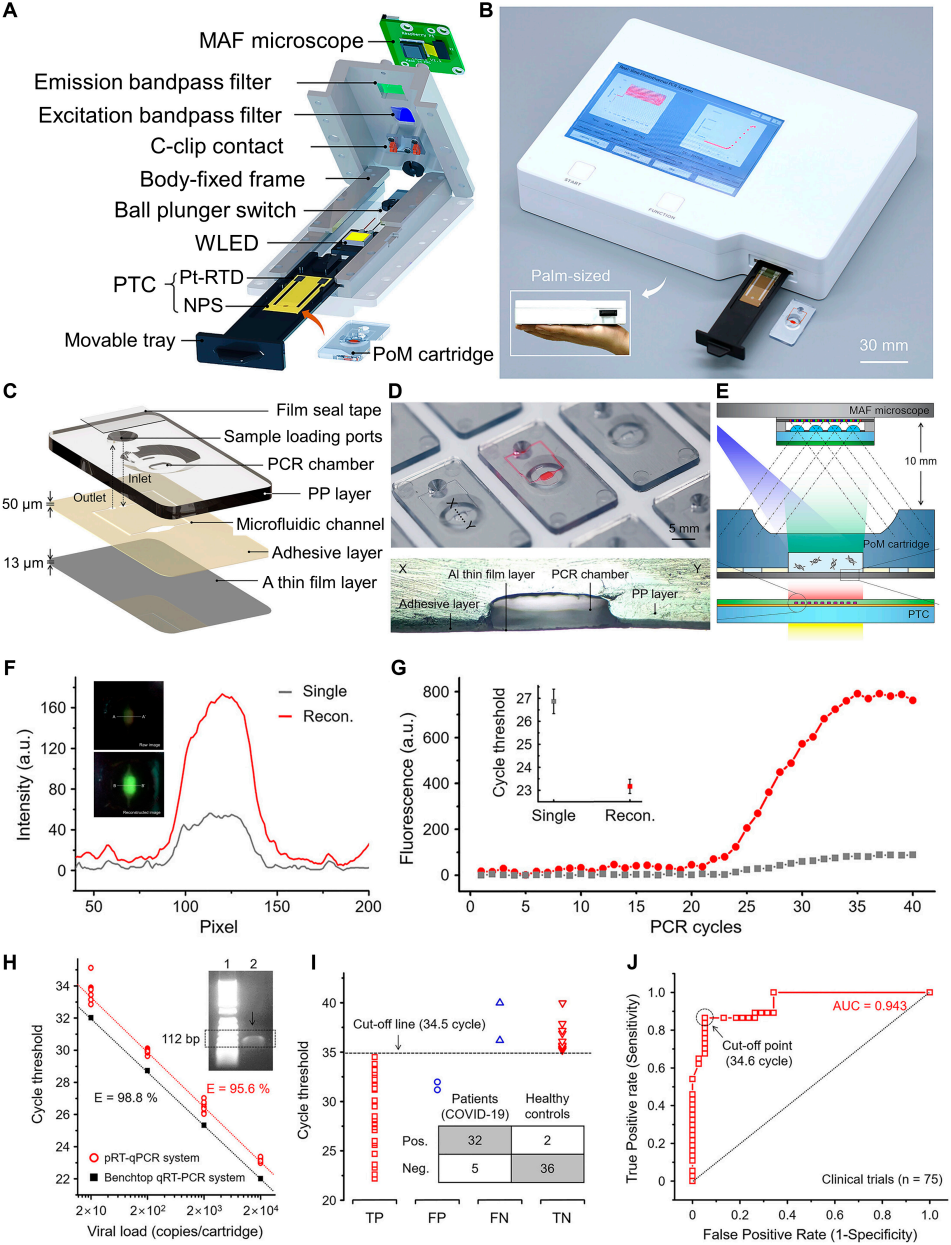
The ultrafast pRT-qPCR system. (A) Schematic diagram of the internal structure of the pRT-qPCR system. (B) Photograph of the fully packaged pRT-qPCR system. (C) Explosion view of the PoM cartridge, consisting of Al thin film, adhesive layer, and polypropylene (PP) layer from botton to top. (D) Optical photograph of the PoM cartridge (top) and the cross-sectional image of the PCR chamber (botton). (E) Schematic diagram of working principle of the pRT-qPCR system. The PTC performs photothermal conversion and heats the reagent in the cartridge through the Al thin film, and the MAF microscope offers fluorescence imaging of the products. (F) Fluorescence images and their intensity profiles of raw single microlens image and reconstructed image. (G) Amplification curves and their corresponding Ct values of the pRT-qPCR before and after the image reconstruction. (H) Comparative analysis of pRT-qPCR system and benchtop qRT-PCR systems from standard curves of the cycle threshold versus the viral concentration. Diagnostic test classification results (I) and ROC curve (J) of 75 clinical samples (37 COVID-19 patients and 38 healthy controls). Reproduced with permission from [241]. Copyright 2023, The Authors, publilshed by American Chemical Society.

Based on previous research, Jeong and coworkers [241] developed an ultrafast plasmonic real-time RT-PCR (pRT-qPCR) system aiming at improving PCR amplification speed and detection efficiency, reducing costs, and simplifying operations. Figure 18A shows the internal structure and primary components of the system, which mainly consists of an ultrafast plasmonic thermocycler (PTC) for rapid photothermal cycling through a white light LED illumination, and an ultrathin microlens array fluorescence (MAF) microscope for high-contrast fluorescence imaging. These functional components are integrated into a palm-sized device through an organized layout. The entire system weighs only 580 g, making it suitable for POCT applications (Fig. 18B). Additionally, a disposable plastic-on-metal (PoM) thin-film cartridge was developed to facilitate plasmonic heating, enabling rapid heat transfer and efficient real-time PCR quantification. As shown in Fig. 18C, the PoM cartridge features a multilayer structure, with the main body being an injection-molded plastic chip. An aluminum thin-film layer, designed for rapid heat conduction, is affixed to the bottom of the chip through an adhesive layer, which has been cut at specific locations to form microfluidic channels. Figure 18D and E shows the photograph of the PoM cartridge and the schematic diagram of its working principle. The PTC achieves a heating rate of 18.85 °C/s and a cooling rate of 8.89 °C/s. The aluminum film, serving as the bottom of the PCR chamber, quickly transfers the heat generated by the underlying PTC to the liquid, enabling rapid and precise thermal cycling control. The MAF microscope is placed above the chip, which can carry out high-contrast fluorescence imaging at short distances and enhancing detection sensitivity and signal-to-noise ratio through image reconstruction algorithms. Figure 18F shows a comparison of the fluorescence images and their fluorescence intensity profiles before and after reconstruction using averaging algorithm, noise subtraction algorithm, and image masking algorithm. The results demonstrated that the image reconstruction algorithm reduced background noise by 98.69%, enhancing the signal-to-noise ratio by 1.33 times. Sensitivity testing with fluorescein isothiocyanate (FITC) dye showed that the LOD of the MAF microscope was 123 nM, which is 2 times higher than that of a conventional fluorescence microscope. As shown in Fig. 18G, when detecting low-concentration samples, MAF microscopy coupled with image reconstruction algorithms can effectively improve the detection limit. The image reconstruction can reduce the Ct value by approximately 4 cycles. The authors applied this system to the diagnosis of COVID-19, which can rapidly diagnose COVID-19 RNA virus within 10 min, achieving an amplification efficiency of 95.6% (Fig. 18H), a classification accuracy of 96.6% in pre-operational tests, and an overall agreement of 91% in clinical diagnostic tests (Fig. 18I and J). This system not only significantly improves molecular diagnostic speed but also demonstrates notable advantages in cost and portability, offering the potential for high-quality on-site testing solutions in public health and primary care settings.

### Prospects of photonic PCR in POCT of nucleic acid

Photonic PCR utilizes compact light sources such as LEDs or lasers as heating elements, eliminating the need for heavy metal thermal blocks for heat conduction and bulky cooling devices for TEC in traditional PCR systems. This gives photonic PCR-based devices significant advantages in terms of miniaturization and low power consumption. Additionally, the ultrafast thermal cycling speed of photonic PCR can greatly reduce the time required for target amplification, aligning well with the demands of POCT of nucleic acid. Consequently, an increasing number of research teams are exploring the development of ultrafast, fully integrated POCT systems for nucleic acid detection based on photonic PCR technology. A primary task in this effort is the development of composite photothermal nanomaterials and integrated detection cartridges (chips) capable of fully automating nucleic acid extraction, enrichment, and purification. The previously mentioned PMNs are an example of effective solutions. During the design of the detection cartridges (chips), targeted optimizations must be made based on the type of photothermal materials used, including considerations for light transmittance, hydrophobicity/hydrophilicity, and high-temperature resistance. Following the purification of nucleic acids and ultrafast amplification of targets, it is necessary to integrate automated detection technologies for the amplification products. Notably, additional steps are often required to eliminate interference from photothermal nanoparticles on the amplification signal. In addition to the mainstream real-time fluorescence PCR and dPCR, colorimetric methods that do not require complex sensing equipment are also emerging, allowing qualitative detection results to be observed directly by the naked eye. In terms of system construction, controlling the photothermal excitation light source is crucial, as the stability of the excitation light directly affects the efficiency of photothermal heating. For result detection part, smartphones with integrated cameras are considered one of the optimal choices, effectively reducing device costs and size [242–245]. With advancements in material synthesis technologies and deeper understanding of photonic PCR mechanisms, it is reasonable to believe that in the near future, integrated ultrafast nucleic acid POCT systems based on photonic PCR will revolutionize on-site rapid nucleic acid testing.

### The commercialization of photonic PCR

Photonic PCR, as an innovative form that disrupts traditional PCR, achieves millisecond-scale temperature switching through photothermal nanomaterials and precise optical excitation, significantly accelerating thermal cycling. It can reduce the typical 2-h PCR amplification time to under 5 min, greatly expanding PCR’s application scope [120]. The global in vitro diagnostic (IVD) market is undergoing rapid growth, with molecular diagnostics being particularly active. In 2022, the global molecular diagnostics market reached 18.4 billion and is projected to reach 37.5 billion by 2026. Consequently, photonic PCR technology holds vast market potential and application prospects.

However, current research on photonic PCR remains in its infancy, with most advances confined to laboratory settings. Its clinical translation still faces several core challenges:

1. Technical bottlenecks require breakthroughs, including (a) enhancing photothermal conversion efficiency and thermal stability of photothermal materials, (b) investigating nanomaterial-induced amplification inhibition and fluorescence quenching mechanisms, (c) developing high-precision temperature monitoring and high-power excitation light control technologies, and (d) integrating automated processing for complex samples and precise detection methods for amplified products.

2. Lack of unified standards and approval frameworks. Variations in photothermal materials, excitation light parameters, optical path designs, and control algorithms across devices compromise result consistency.

3. High clinical application costs. Compared to conventional PCR systems, photonic PCR involves expensive photothermal nanomaterials or consumables, leading to higher per-test costs. Additionally, integration of high-precision components (e.g., high-power lasers) increases equipment expenses.

Multiple research teams are addressing these technical hurdles to accelerate industrialization: designing core–shell structured photothermal nanomaterials to improve thermal stability and reduce PCR inhibition [126,234]; developing medical-grade temperature sensors for real-time fluctuation correction to prevent signal drift; utilizing AI algorithms to eliminate fluorescence background noise and dynamically adjust thresholds for enhanced signal-to-noise ratio [241]; simplifying system integration by designing shared optical paths to reduce hardware complexity, and creating integrated detection chips that combine sample processing, photothermal, and detection units to improve usability [86]; establishing dedicated Ct-value calibration models for photonic PCR to standardize results across devices; and proposing classified approval pathways with dedicated channels for photonic PCR to accelerate regulatory clearance.

With its revolutionary speed, photonic PCR stands at the forefront of molecular diagnostics. Overcoming the 3 key bottlenecks—optical stability, sample compatibility, and cost-effectiveness—is crucial for replacing traditional PCR in scenarios like early cancer screening and emergency infection diagnostics. In the next 3 to 5 years, driven by breakthroughs in core components and material innovation, this technology is poised to achieve commercial adoption in high-demand areas of precision medicine.

## Conclusions and Future Perspectives

The photothermal effect refers to the phenomenon where materials convert light energy into thermal energy based on mechanisms such as plasmon resonance, molecular thermal vibration, or nonradiative relaxation. Due to its extremely high thermal conversion efficiency and ultrafast heating rate, the photothermal effect has attracted significant interest among researchers and has been widely and maturely applied in biomedical fields such as disease treatment and sensing imaging. Inspired by this, in recent years, researchers have explored its application in PCR thermal cycling control, leading to the development of novel ultrafast photonic PCR techniques. Based on working principles, photonic PCR can be primarily categorized into planar-heating photonic PCR based on SPPs of nanofilms and volumetric-heating photonic PCR utilizing the LSPR mechanism of plasmonic nanoparticles. The former’s working principle is similar to traditional PCR, with the key distinction being the replacement of Peltier devices in traditional thermal cyclers with photothermal nanofilms. These nanofilms heat the solution above through light-induced photothermal conversion. In contrast to planar-heating photonic PCR, volumetric-heating photonic PCR directly incorporates nanoparticles into PCR reagent. Under the irradiation of excitation light, the nanoparticles act as microscopic heating units, converting photon energy into heat to directly warm the surrounding solution. Compared to traditional PCR systems, photonic PCR devices do not require bulky heating and cooling modules. The efficient energy conversion allows for ultrafast heating rates with relatively low energy input, making it an ideal choice for POCT of nucleic acid. Photonic PCR technology is gradually demonstrating its potential as the next-generation PCR platform. However, this technology remains in its nascent stage, with numerous challenges requiring resolution.

**Fig. 19:**
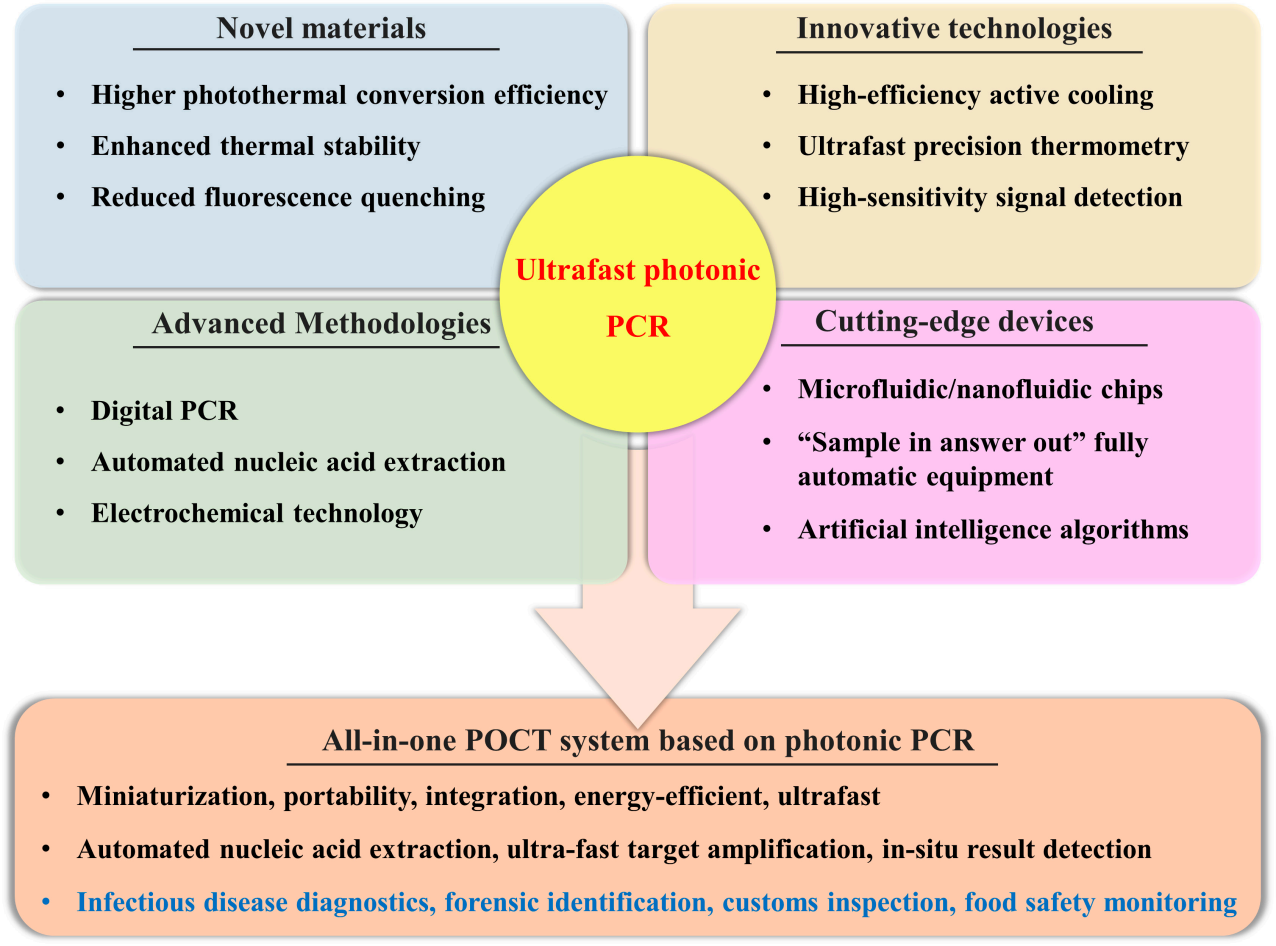
Challenges and future perspectives of photonic PCR.

Here, we propose several highly promising and challenging research directions for photonic PCR (Fig. 19).

1. Development and optimization of novel photothermal materials. Material research primarily encompasses 3 aspects: (a) further enhancing photothermal conversion efficiency to shorten heating durations during thermal cycling and improve PCR amplification efficiency; (b) improving thermal stability to ensure that photothermal materials maintain stable morphology and conversion efficiency throughout thermal cycling, preventing diminished performance due to deformation or aggregation, and avoiding adsorption of PCR reactants that may inhibit amplification; (c) investigating mechanisms of nucleic acid-specific adsorption by photothermal nanomaterials, along with fluorescence signal quenching/enhancement effects. Targeted optimization strategies such as morphology control, surface coating, and concentration adjustment should be implemented to minimize interference with PCR processes and enhance detection sensitivity.

2. Innovative technologies in photonic PCR. In PCR thermal cycling control, most current research only focuses on heating rates, while cooling methods are largely limited to natural convection or fan cooling. There is little research reported on novel active cooling technologies such as nanoparticle-induced active cooling [246–248]. Some microfluidics-integrated approaches achieve ultrafast heating/cooling rates by minimizing reaction volumes. However, this approach does not fundamentally break through the thermal inertia limitations and may compromise detection accuracy. Thus, developing novel active cooling strategies via advanced materials or technologies represents a critical research avenue. Additionally, achieving rapid and precise temperature monitoring is essential for accurate photothermal control. However, conventional thermocouple temperature sensors that measure temperature through contact thermal conduction are inadequate for photothermal processes. Therefore, the development of miniaturized, high-speed, and accurate temperature monitoring technologies will greatly promote precise temperature control in photothermal processes. Lastly, developing specialized detection methods compatible with ultrafast photonic PCR is vital for improving sensitivity, specificity, and accuracy of results.

3. Integration with advanced detection methodologies to develop novel nucleic acid testing technologies suitable for various application scenarios. As previously discussed, combining photonic PCR with dPCR to establish photonic dPCR technology can significantly enhance detection speed and sensitivity. Furthermore, integrating automated nucleic acid extraction upstream enables fully automated sample-to-answer diagnostic workflows. Recent advances in electrochemical sensing for nucleic acid testing offer new perspectives for photonic PCR product detection.

4. Development of cutting-edge devices based on photonic PCR. Synergizing photonic PCR with microfluidic technology could yield compact, portable, and ultra-rapid integrated nucleic acid testing systems. Implementing AI for precision temperature control and amplified product signal detection may catalyze transformative breakthroughs in testing speed and sensitivity.

In recent years, the development of fully integrated POCT systems enabling “sample-to-answer” functionality has emerged as a pivotal direction in nucleic acid detection [249–251]. Photonic PCR employs compact light sources (e.g., LEDs or lasers) as heating elements, eliminating the need for bulky metal thermal conduction and dissipation components found in conventional PCR instruments. Consequently, this technology demonstrates significant advantages in device miniaturization and low power consumption. Furthermore, the ultrafast temperature ramping capabilities of photothermal PCR dramatically reduce target amplification time, which is a critical feature aligning perfectly with POCT requirements. The all-in-one POCT system based on photonic PCR technology integrates automated nucleic acid extraction, ultrafast photothermal target amplification, and in situ detection into a single platform. Leveraging “minute-scale” ultrafast PCR amplification, such system promises “sample-in, answer-out” functionality within 10 min. These integrated nucleic acid testing platforms are poised to revolutionize immediate testing scenarios such as on-site diagnosis of emerging infectious diseases, forensic identification, customs quarantine inspection, food safety monitoring, and so on.
